# Effect of *Lactobacillus fermentum* HFY06 Combined with Arabinoxylan on Reducing Lipid Accumulation in Mice Fed with High-Fat Diet

**DOI:** 10.1155/2022/1068845

**Published:** 2022-04-06

**Authors:** Fang Li, Hui Huang, Yu Zhang, Hongjiang Chen, Xianrong Zhou, Yongpeng He, Xiao Meng, Xin Zhao

**Affiliations:** ^1^Chongqing Collaborative Innovation Center for Functional Food, Chongqing Engineering Research Center of Functional Food, Chongqing Engineering Laboratory for Research and Development of Functional Food, Chongqing University of Education, Chongqing 400067, China; ^2^College of Biological and Chemical Engineering, Chongqing University of Education, Chongqing 400067, China; ^3^Department of Pain Medicine, The Ninth People's Hospital of Chongqing, Chongqing, 400799 Sichuan, China; ^4^College of Food Science, Southwest University, Chongqing 400700, China; ^5^National Teaching Demonstration Center of Food Science and Engineering, Southwest University, Chongqing 400700, China; ^6^Chongqing Key Laboratory of Translational Research for Cancer Metastasis and Individualized Treatment, Chongqing University Cancer Hospital & Chongqing Cancer Institute & Chongqing Cancer Hospital, Chongqing 400030, China; ^7^Department of Public Health, Chengdu University of Traditional Chinese Medicine, Chengdu 611137, China

## Abstract

In this experiment, a high-fat diet was used to induce hyperlipidemia in mice to determine the synergistic effect of AX and *L. fermentum* HFY06 on the prevention of hyperlipidemia and its potential regulatory mechanism. The results of this study showed that after the AX and *L. fermentum* HFY06 synergistic intervention, the body weight, epididymal fat index, blood lipid level, and liver function indexes of mice were improved. In addition, the synbiotics comprising AX and *L. fermentum* HFY06 increased the CAT activity in the serum of mice on a high-fat diet, reduced NO and MDA levels, and improved the body's oxidative stress. From the perspective of molecular biology, on the one hand, AX and *L. fermentum* HFY06 synergistic intervention activated the AMPK pathway to regulate body lipid metabolism; up-regulated the mRNA expressions of CPT-1, PPAR-*α*, CYP7A1, and HSL; and down-regulated the mRNA expressions of ACC, C/EBP*α*, and LPL. On the other hand, the synergistic effect of AX and HFY06 enhanced the mRNA expressions of ZO-1, occludin, and claudin-1 in the small intestine of mice, increased the strength of the intestinal barrier, and optimized the composition of the intestinal microbiota. From the above results, it can be concluded that AX and *L. fermentum* HFY06 have a synergistic effect in improving hyperlipidemia. However, this study was only performed using animal models, and the lipid synthesis and metabolism mechanism are complicated; hence, further clinical studies are needed.

## 1. Introduction

Abnormal fat metabolism or functioning causes an increase in the levels of one or more plasma lipids, and this condition is called hyperlipidemia, which is manifested as hypercholesterolemia, hypertriglyceridemia, or both [1, 2]. These metabolic reactions can accelerate lipid accumulation. In addition, dyslipidemia is also an important cause of fatty liver, cardiovascular- and cerebrovascular-related diseases, high blood sugar, and hypertension [3, 4]. Therefore, dietary methods that reduce hyperlipidemia, lower free fatty acid levels, and inhibit liver lipid synthesis and fat accumulation have aroused the interest of researchers [5, 6].

Dietary fiber is the most important type of compound in cereals, and it has positive effects on the human body, such as improving blood lipid levels, preventing obesity, preventing cardiovascular diseases, preventing colon and rectal cancer, promoting calcium absorption, and improving diabetes symptoms [7]. AX plays a beneficial role in human health, especially its physiological effects of lipid lowering, glucose lowering, and antioxidation, which will become a target for the treatment of hyperlipidemia [8]. Chen et al. found that high fat can cause an increase in fatty acid synthase and acetyl-CoA carboxylase activity, leading to increased TG in mice; after supplementing AX in a high-fat diet, FAS and ACC levels decrease; liver TG decomposition increases; fatty acid oxidation increases; and eventually TG levels return to normal [9].

In the human body, the intestinal flora is a complex microecosystem, in which there are about 300~1000 types of bacteria and the total number is close to 10^13^~1014, mainly including bacteria, archaea, protozoa, fungi, and viruses, which are coevolutionary with the human body [10]. When there is imbalance in the intestinal flora, a large number of gram-negative bacteria will proliferate and produce endotoxins, which will lead to metabolic disorders, obesity, diabetes, and other disorders [11]. Studies have confirmed that adding *Lactobacillus plantarum* LP104 to an animal model of obesity can activate the AMPK/Nrf2/CYP2E1-related pathway and regulate the expression of related proteins, which can effectively improve hyperlipidemia, liver metabolic disorder, and liver oxidative stress response in mice fed with a high-fat diet [12]. It has also been found that the intervention of *L. paracasei* FZU103 in high-fat diet fed mice can reduce the abundance of harmful bacteria and increase the abundance of beneficial bacteria, so as to change the composition of intestinal microbiota to treat hyperlipidemia [13].

Probiotics and AX can improve metabolic diseases, such as obesity, insulin resistance, and hyperlipidemia. However, it is still unclear whether probiotics combined with AX can improve the prevention and treatment effect of hyperlipidemia. However, studies have found that probiotics combined with prebiotics can provide dietary intervention for the treatment of obesity and its related complications. *Bifidobacteria* and *Lactobacillus paracasei*, combined with oat *β*-glucan, can reduce weight gain and metabolic complications by adjusting intestinal short-chain fatty acids and improving intestinal microbial population in high-fat diet fed mice [14]. Tang et al. found that *Lactobacillus plantarum* S58 and barley *β*-glucan synergistically activate the AMPK signal pathway, regulate the expression of genes related to lipid metabolism, improve the structure of the intestinal microbiota, and promote fat metabolism [15]. In this study, AX, which is a nonstarch polysaccharide dietary fiber extracted from wheat. *L. fermentum* HFY06, was isolated from traditional fermented yogurt. In summary, we hypothesized that *L. fermentum* HFY06 combined with AX could adjust fat metabolism and reduce fat accumulation by activating the AMPK signaling pathway; on the other hand, it could regulate the intestinal mucosal barrier function, improve the intestinal flora, and reduce endotoxin production and oxidative stress in mice. To verify this hypothesis, C57BL/6J mice were given *L. fermentum* HFY06 and AX by gavage for 12 weeks. Then, lipid metabolism, AMPK pathway, intestinal barrier, intestinal microbial composition, and oxidative stress were analyzed to determine the potential mechanism and provide a new method for preventing hyperlipidemia.

## 2. Materials and Methods

### 2.1. Source of Materials/Animals

Fifty 6-week-old male C57BL/6J mice were purchased from the animal experiment center of Chongqing Medical University. Basic feed (12.79% fat, 66.67% carbohydrate, and 20.54% protein) and high-fat feed (60.65% fat, 21.22% carbohydrate, and 18.14% protein) were purchased from Jiangsu National Research Center, China. *Lactobacillus fermentum* HFY06 (*L. fermentum* HFY06) was isolated from a unique yak yogurt of Sichuan, China, and is stored in the China General Microbial Culture Collection, Beijing, China (No. 16636). The arabinoxylan (AX) was purchased from Annoron Biotechnology Co., Ltd. (Beijing, China). The purity of AX was greater than 95% (No. 120601b). The ratio of arabinose: xylose is 38: 62.

### 2.2. *L. fermentum* HFY06 Culture and AX Solution

*L. fermentum HFY06* was cultured in de Man, Rogosa, and Sharpe (MRS) medium for 20 h at 37°C. The culture medium was centrifuged at 3000 rpm/min for 10 minutes. The bacterial pellet was then collected, washed twice with sterile saline, and suspended in saline, ready for use. Arabinoxylan was dissolved in distilled water.

### 2.3. Animal Experiments

Fifty C57BL/6J male healthy mice were randomly divided into 5 groups, with 10 mice in each group. The mice were adaptively fed for one week. The feeding conditions were as follows: temperature 25 ± 2°C and light and dark for 12 h, respectively. Group I was fed 10% LFD (48.30% corn flour, 12.00% bran, 17.00% wheat flour, 6.00% soybean meal, 10.00% animal protein, 1.00% lard, and 5.70% premix), with saline as the normal control; group II was fed 60% HFD (18.35% corn flour, 4.56% bran, 6.46% wheat flour, 2.28% soybean meal, 15.30% animal protein, 5.60% sucrose, 28.00% lard, 11.18% whole milk powder, and 8.17% premix), with saline as the model control; group III was fed with the HFD diet, incorporated with AX (200 mg/kg); group IV was fed with the HFD diet incorporated with *L. fermentum* HFY06 (1 × 1010 CFU/kg); and group V was fed with the HFD diet with both *L. fermentum* HFY06 (1 × 1010 CFU/kg) and AX (200 mg/kg). The experiment lasted for 12 weeks, and the weight of mice was weighed and recorded once a week. After the final administration, the mice were allowed to drink freely and fasted for 16-20 h, and their eyeballs were taken to collect blood. The mice were euthanized by cervical dislocation, and liver, epididymal fat, and small intestine tissues were obtained. In addition, the epididymal fat index was analyzed, and the formula for calculating the epididymal fat index was as follows: Epididymal fat index = epididymal fat (g)/mouse body weight (g) × 100. This study has been approved by the Ethics Committee of Chongqing Collaborative Innovation Center for Functional Food (202009124B), Chongqing, China.

### 2.4. Analysis of Serum Lipids

The collected blood samples were placed in the refrigerator for 30 minutes to allow for blood coagulation, and then, they were centrifuged in a 4°C low-temperature centrifuge. Centrifugation conditions were as follows: 3000 r/min, 10 min, and the upper serum was collected. The contents of TC, TG, LDL-C, and HDL-C in mouse serum were determined by the kit (Nanjing Institute of Bioengineering, Nanjing, China).

### 2.5. Analysis of the Antioxidant Index

The serum samples collected in 2.3 were obtained, and the contents of CAT, SOD, NO, and MDA in the serum of mice were detected in each experimental group according to the instructions of the kit (Nanjing Institute of Bioengineering, Nanjing, China).

### 2.6. Analysis of Cytokines

The serum samples collected in 2.3 were obtained, and the contents of IL-6, IL-10, IL-1*β*, IFN-*γ*, and TNF-*α* in mouse serum were determined according to the instructions of the enzyme-linked immunoassay kit (Shanghai Enzyme Biotechnology Co., Ltd., Shanghai, China).

### 2.7. Analysis of Liver Function Indicators

The mouse serum samples were taken out from the refrigerator at -20°C, and the ALT and AST levels were determined in strict accordance with the requirements of the kit (Nanjing Institute of Bioengineering, Nanjing, China).

### 2.8. Pathological Examination of the Liver and Epididymal Adipose Tissue

The liver and epididymal fat tissues of about 1 cm^2^ in size were removed from the newly dissected mice and immersed in 4% formaldehyde solution for fixation for 24 h. Then, dehydration, embedding, sectioning, H&E staining, and finally observation of the pathological section under an optical microscope were performed, and photographs were obtained [16].

### 2.9. Expression of mRNA in the Liver, Epididymal Fat, and Small Intestinal Tissues

The liver and epididymal adipose tissue of mice in each experimental group were obtained to determine the relative expression of key genes in lipid metabolism. The small intestine tissue was removed to determine the relative expression of tight junction proteins (occludin, claudin-1, and ZO-1) between intestinal epithelial cells. The gene sequence is shown in Table 1.

The mouse liver, epididymal fat, and small intestine tissue from were removed from the refrigerator at -80°C, and total RNA was extracted by TRIzol lysate. A nucleic acid analyzer was used to determine the RNA concentration, and the purity value was between 1.8 and 2.0 before subsequent tests could be performed. Then, the RNA was reverse-transcribed into cDNA using a kit (Thermo Fisher Scientific Baltics UAB, Vilnius, Lithuania). Finally, the reverse-transcribed cDNA was used as a template, plus fluorescent dyes, and placed in a quantitative real-time PCR instrument for reaction. The reaction conditions were divided into two steps, referring to the method of Mu et al. [17]. In the experiment, the *β*-actin gene was used as the internal reference gene, the -*ΔΔ*Ct value was calculated, and the 2^-*ΔΔ*Ct^ relative quantification method was used to calculate the expression of each group of genes [17, 18].

### 2.10. Relative Abundance of Selected Gut Bacteria in the Cecal Content

The cecal contents collected in each experimental group were removed from the refrigerator at -80°C, and the total bacterial DNA in the cecal contents of mice was extracted in accordance with the operating requirements of the fecal genomic DNA extraction kit (Tiangen Biotech Co., Ltd., Beijing, China). Using the extracted cecal DNA as a template and the total number of bacteria as an internal reference, polymerase chain reaction (PCR) amplification was carried out [20], and the relative expression levels of *Lactobacillus* sp., *Bifidobacterium* sp., *Firmicutes*, and *Bacteroides* in the intestines of mice in each group were observed. The primer sequences are shown in Table 2.

### 2.11. Data Analysis

The experimental data are expressed as mean ± standard deviation (SD). Graph Pad Prism 7.0 software was used to create graphs. One-way analysis of variance (one-way ANOVA) was used to compare differences between groups, and different letters represented significant differences (*p* < 0.05).

## 3. Results

### 3.1. Effect of AX Combine *L. fermentum* HFY06 on Body Weight and Epididymal Fat Index

**Figure 1:**
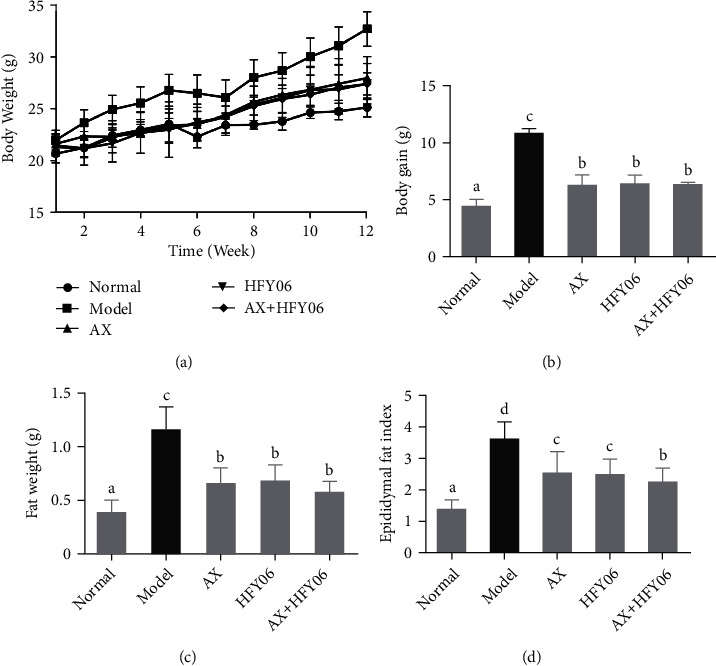
Effect of AX combine *L. fermentum* HFY06 supplementation on (a) body weight curve; (b) weight gain; (c) epididymal fat weight; and (d) epididymal fat index. AX: high-fat diet + AX (200 mg/kg); HFY06: high-fat diet + *L. fermentum* HFY06 (1.0 × 10^10^ CFU/kg); AX+HFY06: high-fat diet + AX (200 mg/kg) + *L. fermentum* HFY06 (1.0 × 10^10^ CFU/kg). Data are means ± SD (*n* =10 per group). ^a–c^Mean values with different letters in the same column are significantly different (*p* < 0.05) according to Duncan's multiple range test.

During the gavage period, the weight changes in mice are recorded every week, and the results are shown in Figure 1(a) and 1(b)). The body weight of mice did not change significantly before the experiment. After the 12-week experiment, the body weight of mice in the high-fat model group was significantly different from that of mice in the normal group (*p* < 0.05), and the body weight of mice in the model group was 28% higher than that of mice in the normal group. The body weight of mice in AX group, HFY06 group, and AX+HFY06 group decreased by 12.59%, 14.31%, and 14.34%, respectively. In addition, the weight of the epididymal fat and epididymal fat index was analyzed after the mice were dissected. The results are shown in Figure 1(c) and 1(d). The weight of epididymal fat in the model group was the highest, reaching 1.15 g, and the average weight of epididymal fat in the normal group was 0.37 g. After treatment with AX or *L. fermentum* HFY06, and AX and HFY06 intervention, the epididymal fat weight of mice was reduced, but no significant weight differences were found between the three intervention groups (*p* < 0.05). This result indicated that AX and HFY06 intervention had inhibitory effects on weight gain and fat accumulation in mice.

### 3.2. Effect of AX Combine *L. fermentum* HFY06 on Serum Lipid Parameters in Mice

After 12 weeks of continuous intervention, the serum levels of TG, TC, LDL-C, and HDL-C were measured. The results are shown in Table 3. Compared with the normal group, the contents of TG, TC, and LDL-C were significantly increased in the model group (*p* < 0.05) by 84.61%, 64.62%, and 145.16%, respectively, and the content of HDL-C was significantly decreased (*p* < 0.05) by 30.30%, which indicated that the high-fat diet successfully induced dyslipidemia in mice. After the intervention of AX and *L. fermentum* HFY06, dyslipidemia was improved to varying degrees. Compared with the model group, AX could significantly reduce the contents of TG and TC in the serum, but it had no significant effect on the level of LDL-C. *L. fermentum* HFY06 significantly reduced the contents of TG, TC, and LDL-C in the serum and increased the content of HDL-C. However, there was no significant difference in the effect of improving blood lipid levels between the *L. fermentum* HFY06 group and the AX+HFY06 synergistic group.

### 3.3. Effect of AX Combine *L. fermentum* HFY06 on Serum AST and ALT Activity in Mice

**Figure 2:**
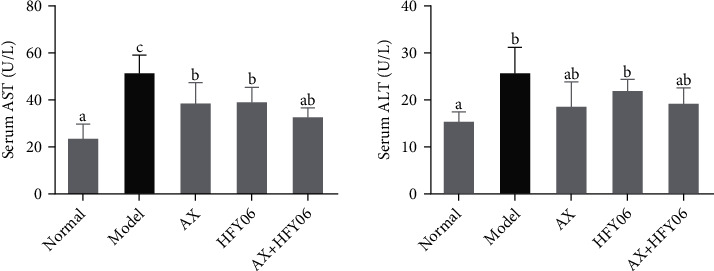
Effect of AX combine *L. ermentum* HFY06 on serum ALT and AST levels in high-fat diet mice. AX: high-fat diet + AX (200 mg/kg); HFY06: high-fat diet + *L. fermentum* HFY06 (1.0 × 10^10^ CFU/kg); AX+HFY06: high-fat diet + AX (200 mg/kg) + *L. fermentum* HFY06 (1.0 × 10^10^ CFU/kg). Different letters above the values indicate significant differences (*p* < 0.05). Data are means ± SD (*n* =10 per group). ^a–c^Mean values with different letters in the same column are significantly different (*p* < 0.05) according to Duncan's multiple range test.

ALT and AST, important amino acid transaminases in the body, are markers that reflect liver cell damage [21]. After 12 weeks of modeling and intragastric intervention, the activities of AST and ALT in the serum of mice in all experimental groups changed to varying degrees (Figure 2). Compared with mice in the normal group, the ALT and AST levels were significantly increased in the serum of mice in the model group (*p* < 0.05), indicating that the high-fat diet caused a certain degree of damage to the liver of mice. AX and HFY06 can effectively inhibit high-fat diet-induced ALT and AST elevations and alleviate liver damage. However, the combined effect of AX and HFY06 was not superior to that alone.

### 3.4. Effect of AX Combine *L. fermentum* HFY06 on Antioxidation Parameters in Mice

After 12 weeks of continuous intervention in mice, the serum levels of CAT, SOD, NO, and MDA in mice in all experimental groups are determined, as shown in Table 4. High-fat diet caused a decrease in CAT and SOD enzyme activities and an increase in NO and MDA levels in the serum of mice. This showed that high-fat diet caused a certain degree of oxidative stress damage in mice. After the intervention of AX, *L. fermentum* HFY06, and AX+HFY06, the activity of antioxidant enzymes CAT and SOD was increased in the serum, but the three intervention methods had no significant effect. On the other hand, AX+HFY06 could significantly inhibit the increase in NO and MDA contents caused by the high-fat diet. The synergistic effect of AX and HFY06 was better than that of AX and *L. fermentum* HFY06 alone.

### 3.5. Effect of AX Combine *L. fermentum* HFY06 on Serum Cytokine Levels in Mice

After 12 weeks of continuous intervention, the levels of cytokines in the serum of mice in all experimental groups are determined, as shown in Table 5. The levels of serum proinflammatory factors IL-6, IFN-*γ*, TNF-*α*, and IL-1*β* in the model group were the highest, while the level of the anti-inflammatory factor IL-10 was the lowest. After AX or *L. fermentum* HFY06, and AX and HFY06 synergistic intervention, the levels of IL-1*β*, TNF-*α*, IFN-*γ*, and IL-6 were significantly reduced, and the content of IFN-*γ* was even lower than that in the normal group. In addition, the AX and HFY06 synergistic group showed reduced content of TNF-*α*, and the effect of AX and HFY06 combination was significantly greater than that of AX alone and *L. fermentum* HFY06 alone (*p* < 0.05). The AX and HFY06 synergistic group also showed a significantly increased level of anti-inflammatory factor IL-10, which was better than that in the AX alone group. This showed that AX and *L. fermentum* HFY06 could increase the level of anti-inflammatory factor IL-10 and reduce the levels of proinflammatory factors TNF-*α*, IFN-*γ*, and IL-6 in the serum, thereby exerting a certain regulatory effect on blood lipid levels in hyperlipidemic mice.

### 3.6. Effect of AX Combine *L. fermentum* HFY06 on Histopathological in Mice

**Figure 3:**
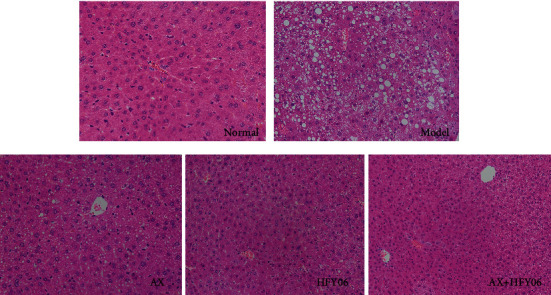
H&E pathological observation of liver tissue in mice. Magnification 20×. AX: high-fat diet + AX (200 mg/kg); HFY06: high-fat diet + *L. fermentum* HFY06 (1.0 × 1010 CFU/kg); AX+HFY06: high-fat diet + AX (200 mg/kg) + *L. fermentum* HFY06 (1.0 × 10^10^ CFU/kg).

As shown in Figure 3, the liver tissue of mice in the normal group did not show fatty degeneration or fat infiltration, rich cytoplasm of liver cells, neatly arranged, large and normal nuclei in the center, and clear tissue structure. Hepatocytes of mice in the model group induced by high-fat diet were swollen and showed obvious steatosis. Most of the hepatocytes contained lipid droplets, and some of the lipid droplets were larger and showed a regular round shape. After AX, *L. fermentum* HFY06, and AX and HFY06 synergistic intervention, the fat droplets in liver cells were altered. In the AX group, the cytoplasm still showed some large lipid droplets, but the number was significantly reduced. In the *L. fermentum* HFY06 group, the number of lipid droplets was less, and the number and density of fat droplets in the AX+HFY06 group were less, and fatty degeneration in the liver was significantly improved. This indicated that AX and HFY06 synergistically improved liver lipid accumulation and prevented the absorption of fat in the body.

**Figure 4:**
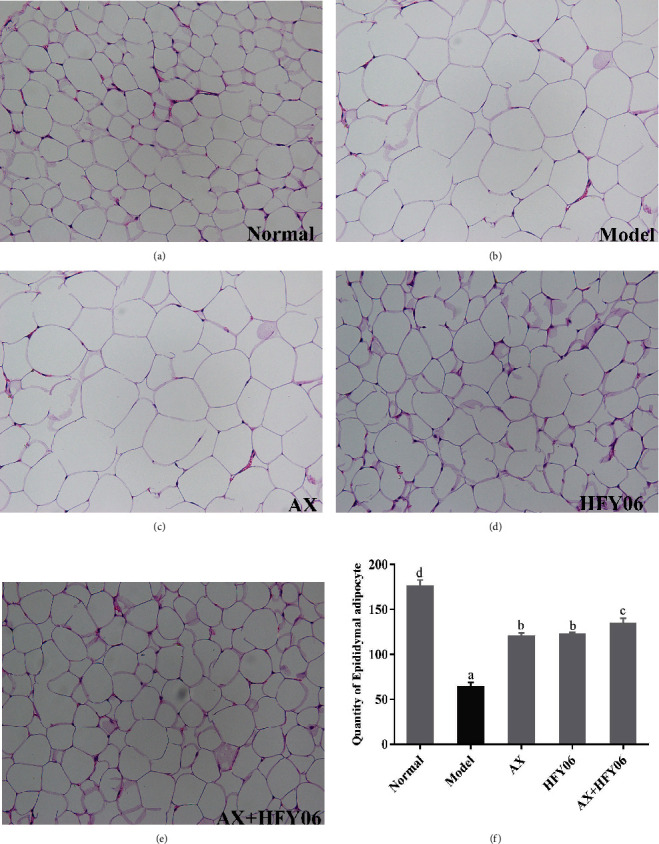
(a–e) H&E pathological observation of epididymal fat in mice. Magnification 20×. (f) Quantity of epididymal adipocyte. AX: high-fat diet + AX (200 mg/kg); HFY06: high-fat diet + *L. fermentum* HFY06 (1.0 × 10^10^ CFU/kg); AX+HFY06: high-fat diet + AX (200 mg/kg) + *L. fermentum* HFY06 (1.0 × 10^10^ CFU/kg). Data are means ± SD (*n* =6 per group). (a–d) Mean values with different letters in the same column are significantly different (*p* < 0.05) according to Duncan's multiple range test.

As shown in Figure 4, the fat cells in the normal group are uniform, neatly arranged, and dense. Fat cells of mice in the model group were hypertrophied and loosely arranged. Some fat cells were even three or four times larger than those in mice in the normal group. After 12 weeks of drug intervention, the arrangement of adipocytes became more compact and the cell volume became smaller. In particular, the synergistic effect of AX and HFY06 significantly reduced adipocyte hypertrophy.

### 3.7. Effect of AX Combine *L. fermentum* HFY06 on Expression of Related Lipid Metabolism mRNA in the Epididymal Adipose Tissue of Mice

**Figure 5:**
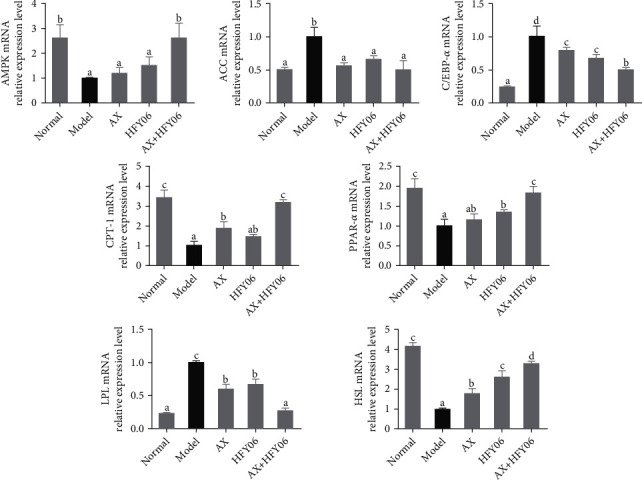
The lipid metabolism mRNA expression in epididymal fat tissue of mice. AX: high-fat diet + AX (200 mg/kg); HFY06: high-fat diet + *L. fermentum* HFY06 (1.0 × 10^10^ CFU/kg); AX+HFY06: high-fat diet + AX (200 mg/kg) + *L. fermentum* HFY06 (1.0 × 10^10^ CFU/kg). Data are means ± SD (*n* =6 per group). (a–e) Mean values with different letters in the same column are significantly different (*p* < 0.05) according to Duncan's multiple range test.

The relative expressions of AMPK, ACC, CPT-1, C/EBP-*α*, LPL, PPAR-*α*, and HSL genes in the epididymal adipose tissue are determined by the qPCR method, and the results are shown in Figure 5, and the melting curves are shown in Figure S1. Compared with the normal group, the levels of AMPK, CPT-1, PPAR-*α*, and HSL were significantly reduced in the model group (*p* < 0.05), while the levels of ACC, C/EBP-*α*, and LPL were increased. After AX, *L. fermentum* HFY06, and AX and HFY06 synergistic intervention, lipid synthesis, transport, and decomposition were improved, which significantly reversed the gene expression trend of model mice. AX or *L. fermentum* HFY06 significantly inhibited the down-regulation of AMPK, CPT-1, HSL, and PPAR-*α* expression, as well as the up-regulation of ACC, C/EBP-*α*, and LPL, and the synergistic regulation effect of AX and HFY06 was better than the regulation effect of AX alone or *L. fermentum* HFY06 alone (*p* < 0.05).

### 3.8. Effect of AX Combine *L. fermentum* HFY06 on Expression of Related Lipid Metabolism mRNA in the Liver Tissue of Mice

**Figure 6:**
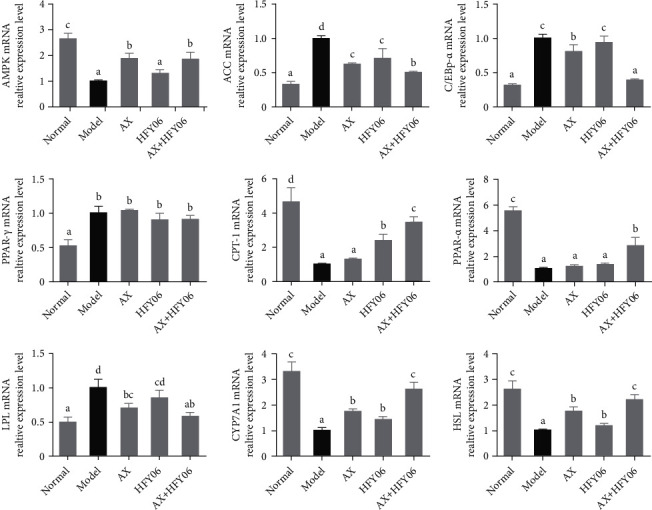
The lipid metabolism mRNA expression in liver tissue of mice. AX: high-fat diet + AX (200 mg/kg); HFY06: high-fat diet + *L. fermentum* HFY06 (1.0 × 10^10^ CFU/kg); AX+HFY06: high-fat diet + AX (200 mg/kg) + *L. fermentum* HFY06 (1.0 × 10^10^ CFU/kg). Data are means ± SD (*n* =6 per group). (a–d) Mean values with different letters in the same column are significantly different (*p* < 0.05) according to Duncan's multiple range test.

This study determines the mRNA expression of genes involved in fat synthesis, fatty acid *β* oxidation, TG breakdown, and cholesterol metabolism in the AMPK-ACC signaling pathway in liver tissues (Figure 6), and the melting curves are shown in Figure S2. Compared with the normal group, the relative expression of the AMPK gene was significantly reduced in the liver tissue of mice in the model group (*p* < 0.05). After AX, *L. fermentum* HFY06, and AX and HFY06 synergistic intervention, the expression of AMPK was increased, especially the synergistic effect of AX+HFY06 was better than that of *L. fermentum* HFY06 alone. In addition, the high-fat diet increased the expression of fatty acid synthesis-related genes (ACC, C/EBP-*α*, and PPAR-r) in mice. After AX and HFY06 synergistic intervention, the expressions of ACC and C/EBP-*α* were significantly down-regulated, and the effect of down-regulation was better than that of AX and *L. fermentum* HFY06 alone, but these three intervention methods had no significant regulatory effect on the relative expression of the PPAR-*γ* gene. At the same time, high-fat diet resulted in the down-regulation of fatty acid *β*-oxidation related genes (PPAR-*α* and CPT-1). After 12 weeks of co-intervention with AX+HFY06, the expressions of PPAR-*α* and CPT-1 were significantly up-regulated, and the effect was better than AX or *L. fermentum* HFY06 alone. The high-fat diet reduced the expression of bile acid synthesis CYP7A1 gene in mice, increased the expression of CYP7A1 after supplementation of AX+HFY06, and promoted the conversion of cholesterol into bile acids. In addition, the synergistic effect of AX+HFY06 increased the mRNA expressions of HSL, decreased the mRNA expressions of LPL, and promoted the hydrolysis of TGs into fatty acids.

### 3.9. Effect of AX Combine *L. fermentum* HFY06 on Expression of Tight Junction Protein mRNA in the Small Intestine of Mice

**Figure 7:**
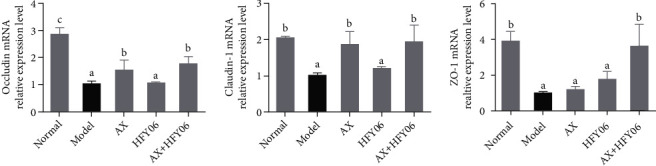
The claudin-1, occludin, and ZO-1 mRNA expression in small intestine tissue of mice. AX: high-fat diet + AX (200 mg/kg); HFY06: high-fat diet + *L. fermentum* HFY06 (1.0 × 10^10^ CFU/kg); AX+HFY06: high-fat diet + AX (200 mg/kg) + *L. fermentum* HFY06 (1.0 × 10^10^ CFU/kg). Data are means ± SD (*n* =6 per group). (a–c) Mean values with different letters in the same column are significantly different (*p* < 0.05) according to Duncan's multiple range test.

High-fat diet had a negative regulatory effect on the intestinal mucosa, increasing the bacteria in the intestinal flora that destroy the barrier, thereby destroying the integrity of the barrier. It can be seen from Figure 7 that high-fat diet reduced the expression of tight junction proteins occludin, claudin-1, and ZO-1 in the small intestine, indicating that high-fat diet had a destructive effect on the intestinal mucosal barrier. The combined effect of AX and HFY06 could significantly increase the expression of ZO-1, and the synergistic effect of AX+HFY06 was better than the single effect of AX and HFY06. AX and AX+HFY06 had the same up-regulation effect on the mRNA expressions of occludin and claudin-1, and the up-regulation was higher than that with *L. fermentum* HFY06 alone.

### 3.10. Effect of AX Combine *L. fermentum* HFY06 on Relative Abundance of Selected Gut Bacteria in the Cecal Content of Mice

**Figure 8:**
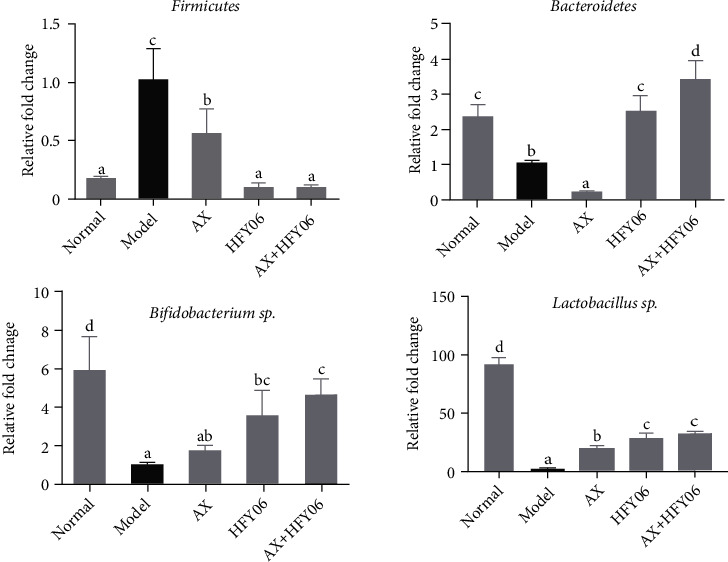
Effect of AX combine *L. fermentum* HFY06 supplementation on abundances of *Firmicutes*, *Bacteroidetes*, *Bifidobacterium* sp., and *Lactobacillus* sp. AX: high-fat diet + AX (200 mg/kg); HFY06: high-fat diet + *L. fermentum* HFY06 (1.0 × 10^10^ CFU/kg); AX+HFY06: high-fat diet + AX (200 mg/kg) + *L. fermentum* HFY06 (1.0 × 10^10^ CFU/kg). Data are means ± SD (*n* =6 per group). (a–d) Mean values with different letters in the same column are significantly different (*p* < 0.05) according to Duncan's multiple range test.

Using the total number of bacteria as an internal reference, the relative expression results of *Firmicutes*, *Bacteroides*, *Lactobacillus* sp., and *Bifidobacterium* sp. in the feces of mice in all groups are shown in Figure 8. High-fat diet changed the composition of intestinal bacteria, increased the abundance of *Firmicutes*, and reduced the abundances of *Bacteroides*, *Lactobacillus* sp., and *Bifidobacterium* sp. Compared with the normal group, the levels of *Firmicutes*, *Bacteroides*, *Lactobacillus* sp., and *Bifidobacterium* sp. in the high-fat diet group were 5.94 times, 0.44 times, 0.01 times, and 0.16 times that of the normal diet group. However, after AX, *L. fermentum* HFY06, and AX +HFY06 synergistic intervention, the structure of the intestinal bacterial flora was altered. The *L. fermentum* HFY06 group and the AX+HFY06 group showed significantly reduced abundance of *Firmicutes*, and this effect was significantly better than that of AX alone. Compared with the high-fat diet group, especially the AX+HFY06 synergistic group, showed increased abundances of *Bacteroides*, *Lactobacillus* sp., and *Bifidobacterium* sp. The expression levels of *Bacteroides*, *Lactobacillus* sp., and *Bifidobacterium* sp. were 3.29 times, 32.22 times, and 4.61 times that of model group.

## 4. Discussion

Hyperlipidemia is a global epidemic, which can lead to a high incidence of cardiovascular diseases [21]. Hyperlipidemia is also a metabolic syndrome, with multiple lipid characteristics, such as hypercholesterolemia, hypertriglyceridemia, and familial combined hyperlipidemia, and it may have a great adverse effect on health [22]. With regard to the current hot spots in the treatment of hyperlipidemia, researchers tend to find suitable natural products or probiotics to prevent or reduce the harm caused by hyperlipidemia. In this study, an animal model of hyperlipidemia was established through the induction of a high-fat diet for 12 consecutive weeks, and AX and HFY06 were synergistically used for intervention in hyperlipidemic mice. The mice were analyzed for antioxidant, anti-inflammatory, and lipid metabolism mRNA.

High-fat diet will not only cause weight gain, visceral obesity, and other symptoms, but it will also cause abnormal metabolism of blood lipids. In severe cases, hyperlipidemia may occur. The levels of LDL-C, HDL-C, TC, and TG blood lipids and ALT and AST enzyme activities are often used as the clinical criteria for the diagnosis of hyperlipidemia [23]. High-fat diet significantly increased the weight of mice. The weight of mice in the high-fat diet group was 28% higher than that of mice in the normal group. The serum TC, TG, and LDL-C levels of mice in the high-fat diet model group were significantly higher than those of mice in the normal diet group, while the serum HDL-C level was significantly lower than that in the normal diet group. The results of this experiment are consistent with the symptoms of hyperlipidemia reported by related studies: hypercholesterolemia, hypertriglyceridemia, low HDL-C levels, and high LDL-C levels [24]. After intragastric intervention with AX and *L. fermentum* HFY06, the mice lost weight, the weight of epididymal fat tissue was reduced, and the fat cells became smaller and were arranged more regularly. At the same time, the contents of TG and TC in the serum were decreased, and the content of HDL-C was increased. In addition, hyperlipidemia is accompanied by obesity, and lipids tend to accumulate in liver cells. It could be seen from the results of liver H&E staining that there were more lipid droplets and fatty vesicles in the liver of mice in the model group. However, the synergistic effect of AX and HFY06 significantly reduced the number and density of lipid droplets in liver tissues, and it could effectively inhibit the increase in ALT and AST levels caused by high-fat diets and alleviate liver damage. However, for TG and TC, the experimental results show that the combined effect of AX and HFY06 is not better than that of AX or HFY06 alone, which may be due to the short experimental period, and the combined group has not produced a more significant effect on TG and TG in the blood.

Dyslipidemia is the main risk factor for cardiovascular disease. In the past few years, increasing evidence has shown that dyslipidemia is closely related to oxidative stress [25–27]. When dyslipidemia occurs in the body, the oxidation/reduction balance in the body is disturbed, and a large amount of reactive oxygen species (ROS) is produced. ROS can react with various cellular components, such as membrane phospholipids, proteins, and nucleic acids, and it can cause cell structure damage [28]. Under normal physiological conditions, the body has a series of antioxidants that can maintain the body's oxidation/reduction balance, mainly including SOD, GSH, and CAT. SOD is an important antioxidant metal enzyme, which can catalyze the rapid conversion of superoxide anion (O2^−^) into hydrogen peroxide (H_2_O_2_), and then, CAT converts H_2_O_2_ into H_2_O and O_2_, thereby scavenging oxygen-free radicals [29]. MDA is the product of lipid peroxidation between free radicals and polyvalent unsaturated fatty acids in phospholipids. When membrane lipids are under oxidative stress, the production of MDA is increased [30]. Therefore, MDA content and SOD activity can reflect the body's oxidative stress damage level and the ability to defend against free radical damage. In the current study, after the intervention of AX, *L. fermentum* HFY06, and AX+HFY06, the activity of antioxidant enzymes CAT and SOD was increased in the serum. On the other hand, AX+HFY06 could significantly reduce the contents of NO and MDA in the serum of high-fat diet fed mice, and the effect was better than that of AX and *L. fermentum* HFY06 alone.

Inflammation is a series of complex physiological and pathological reactions produced by the body to harmful stimuli from the internal and external environments. It is a protective defense response and a cause of many diseases in the human body, including oxidative stress, fatty liver, vascular diseases, diabetes, and obesity [31, 32]. Tahara et al. found that patients with diabetes and obesity have accompanying low levels of inflammation and insulin resistance [33]. A study has also found that in people with simple hyperlipidemia characterized by an increase in blood lipid levels, the levels of serum inflammatory factors are also increased, indicating that patients with simple hyperlipidemia are already in a state of inflammation [34]. In this study, after AX, *L. fermentum* HFY06, and AX and HFY06 synergistic intervention, the levels of proinflammatory factors IL-1*β*, TNF-*α*, IFN-*γ*, and IL-6 were significantly reduced in the serum of high-fat diet fed mice, while the level of the anti-inflammatory factor IL-10 was increased. In addition, AX and HFY06 synergistically reduced the content of TNF-*α*, and its effect was significantly greater than that of AX and *L. fermentum* HFY06 alone (*p* < 0.05). TNF-*α* interferes with and hinders the metabolism of TGs and cholesterol, thereby further affecting the body's lipid metabolism, and ultimately achieving the effect of regulating blood lipids to a certain extent [35]. Comprehensive experimental results have shown that AX and *L. fermentum* HFY06 can regulate the body's inflammatory state, accelerate the metabolism of TGs and cholesterol, and reduce lipid deposition, thereby exerting a certain therapeutic effect on hyperlipidemia and obesity in hyperlipidemic mice.

Although the experimental results show that AX and *Lactobacillus fermentum* HFY06 can alleviate the elevated blood lipids caused by high-fat diet, and improve the level of inflammation and oxidative stress in the body, the combined effect of AX and HFY06 was not better than the effect of single effect. It may be that the intervention time is insufficient to control the parameters of the disease, or the dose of AX combined with HFY06 is inappropriate, and the excessive concentration of the intervention may also increase the metabolic burden of the mice. Further studies are needed to study the combined effect of different doses of AX and HFY06 on the improvement of fat accumulation in high-fat diet mice.

The liver is an important place for the body's energy metabolism. When the synthesis, transport, and decomposition of fatty acids and triglycerides in the liver are hindered, lipids may be deposited in the liver and gradually form fatty liver. In this study, changes in the synthesis and decomposition pathways of lipids in the liver of mice were examined. Experiments suggest that AX and HFY06 can improve the lipid metabolism disorder in mice, which may be achieved through the following ways: (1) AX combined with arabinoxylan can inhibit the synthesis of fat: ACC is the downstream target molecule of AMPK. Activation of AMPK can lead to its phosphorylation and inactivation, which will inhibit the expression of ACC and reduce the synthesis of fatty acids and cholesterol [36, 37]. Yuan et al. found that aged oolong tea reduces fat accumulation and dyslipidemia caused by high-fat diet by regulating the AMPK/ACC signaling pathway [38]. (2) C/EBP*α*-mediated lipid synthesis: PPAR-*γ* and C/EBP-*α* are transcription factors that regulate adipocyte differentiation to produce triglycerides. As adipocytes differentiate, adipose tissue accumulates and the contents of PPAR-*γ* and C/EBP-*α* increase [39]. (3) PPAR-*α*-mediated fatty acid oxidation metabolism: PPAR-*α* plays an important role in the oxidative metabolism of lipids. It can activate the lipid oxidation gene CPT-1, promote the decomposition of lipids, and reduce the accumulation of lipids in the body [40]. LPL is related to fatty acid intake and is regulated by PPAR-*γ*. The LPL expression is low, and less fat is synthesized [41]. AMPK can also increase the phosphorylation level of HSL, activate it, stimulate the oxidation of fatty acids, and accelerate lipolysis in the adipose tissue [42]. In this study, high-fat diet fed mice synthesized fat by up-regulating C/EBPa and PPAR-*γ* mRNA expressions, reducing the mRNA expressions of PPAR-*α* and CPT-1, inhibiting the *β*-oxidation of fat, reducing the relative expression of HSL, and inhibiting TGs, which caused weight gain, fat accumulation, and abnormal blood lipids in mice. However, the supplementation of AX+HFY06 significantly reversed this phenomenon, down-regulating the relative mRNA expressions of C/EBPa and LPL, inhibiting fat synthesis, up-regulating the relative mRNA expressions of CPT-1 and PPAR-*α*, and promoting fat *β*-oxidation. The AX and HFY06 synergistic effect was greater than that of AX and *L. fermentum* HFY06 alone. CYP7A1 is the rate-limiting enzyme that converts cholesterol into bile acids in the liver [43]. The mRNA expression of CYP7A1 was inhibited in mice in the high-fat diet group, but the expression of CYP7A1 was up-regulated after supplementation with AX+HFY06, which accelerated the process of conversion and excretion from cholesterol to bile acids.

Intestinal barrier dysfunction is related to metabolic diseases, including imbalance of intestinal flora, destruction of intestinal mucosal integrity, increased intestinal mucosal permeability, excessive proliferation of small intestinal bacteria, and decreased intestinal tight junction proteins [44]. A study on hyperlipidemic Sprague-Dawley (SD) rats also confirmed that the mRNA expressions of ZO-1 and occludin were decreased in the intestine, and the permeability of the intestinal mucosa was enhanced [45]. Jiang et al. also found that the expressions of intestinal tight junction proteins, such as ZO-1, claudin-1, and occludin, were decreased, and the permeability of the intestinal mucosa was increased, leading to a large number of intestinal LPS entering the body circulation through the intestine, while the increase in circulating LPS can cause oxidative stress in the body [46]. This is consistent with the results of this study. The activities of SOD and CAT in high-fat diet fed mice were reduced. At the same time, the mRNA expressions of ZO-1, occludin, and claudin-1 were significantly reduced in the small intestine of mice in the model group, which led to increased intestinal permeability. In addition, there were more *Firmicutes* and fewer *Bacteroides*, *Lactobacillus* sp., and *Bifidobacterium* sp. in the intestinal microbes of high-fat diet fed mice. After the combined intervention of AX and HFY06, the mRNA expressions of ZO-1, occludin, and claudin-1 were increased; the intestinal mucosal barrier damage caused by high-fat diet was improved; the abundances of *Lactobacillus* sp., *Bifidobacterium* sp., and *Bacteroides* were increased; the abundance of *Firmicutes* was reduced; and the composition of the intestinal microbiome was effectively optimized. AX combined with *L. fermentum* HFY06 can regulate the intestinal mucosal barrier function and improve the composition of intestinal microorganisms, thereby regulating the genes related to liver fatty acid metabolism, including reducing the expression of synthetic fat genes C/EBP-*α* and LPL; increasing the expression of lipolytic genes CPT-1, PPAR-*α*, and HSL; and regulating the expression of CYP7A1 and promoting cholesterol absorption and excretion. AX combined with *L. fermentum* HFY06 can improve hyperlipidemia in mice, which may be directly or indirectly related to these targets, but lipid metabolism in the body is a multifactor, multilink, and multitarget complex process. Therefore, the specific mechanism of AX combined with *L. fermentum* HFY06 to improve hyperlipidemia still needs further research.

## 5. Conclusions

This study demonstrated for the first time that a synbiotic comprising AX and *L. fermentum* HFY06 improved lipid accumulation in high-fat diet fed mice. AX and HFY06 synergistically inhibit fat accumulation mainly through (1) increasing the rich intestinal flora, while promoting the growth of beneficial intestinal flora and improving the intestinal barrier effect. (2) Down-regulate the expression of ACC, C/EBP*α*, and LPL, and reduce fat synthesis. Up-regulate the expression of PPAR-*α* and CPT-1 genes and increase lipid oxidation. Increase the expression of CYP7A1 and accelerate the conversion and excretion of cholesterol to bile acids. *Lactobacillus fermentum* HFY06 and AX, as components of functional foods, have high application prospects in regulating lipid metabolism and preventing obesity. However, this study was only carried out using animal models, and the lipid synthesis and metabolism mechanism is complicated. Hence, further clinical studies are needed.

## Figures and Tables

**Table 1:** Primer sequences used in the quantitative real-time PCR for the liver, epididymal fat, and small intestine tissue gene expression analysis [19].

Gene names	Primer sequence
AMPK	Forward: 5′-GTCAAAGCCGACCCAATGATA-3′
	Reverse: 5′-CGTACACGCAAATAATAGGGGTT-3′

ACC	Forward: 5′-AGTGATGGTGGCCTGCTCTTG-3′
	Reverse: 5′-AGCAGACGGTGAGCGCATTA-3′

C/EBP*α*	Forward: 5′-GCGGGAACGCAACAACATC-3′
	Reverse: 5′-GTCACTGGTCAACTCCAGCAC-3′

PPAR-*γ*	Forward: 5′-AGGCCGAGAAGGAGAAGCTGTTG-3′
	Reverse: 5′-TGGCCACCTCTTTGCTGTGCTC-3′

CPT-1	Forward:5′-TGGCATCATCACTGGTGTGTT-3′
	Reverse: 5′-GTCTAGGGTCCGATTGATCTTTG-3′

PPAR-*α*	Forward: 5′-AACATCGAGTGTCGAATATGTGG-3′
	Reverse: 5′-CCGAATAGTTCGCCGAAAGAA-3′

LPL	Forward: 5′-TTGCCCTAAGGACCCCTGAA-3′
	Reverse: 5′-TTGAAGTGGCAGTTAGACACAG-3′

HSL	Forward: 5′-GATTTACGCACGATGACACAGT-3′
	Reverse: 5′-ACCTGCAAAGACATTAGACAGC-3′

CYP7A1	Forward: 5′-GCTGTGGTAGTGAGCTGTTG-3′
	Reverse: 5′-GTTGTCCAAAGGAGGTTCACC-3′

Claudin-1	Forward: 5′-GGGGACAACATCGTGACCG-3′
	Reverse: 5′-AGGAGTCGAAGACTTTGCACT-3′

Occludin	Forward: 5′-TGAAAGTCCACCTCCTTACAGA-3′
	Reverse: 5′-CCGGATAAAAAGAGTACGCTGG-3′

ZO-1	Forward: 5′-GCCGCTAAGAGCACAGCAA-3′
	Reverse: 5′-TCCCCACTCTGAAAATGAGGA-3′

*β*-actin	Forward: 5′-CATGTACGTTGCTATCCAGGC-3′
	Reverse: 5′-CTCCTTAATGTCACGCACGAT-3′

**Table 2:** Primer sequences used in the quantitative real-time PCR for cecal content gene expression analysis [19].

Organism names	Primer sequence
*Total bacteria*	Forward:5′-ACTCCTACGGGAGGCAGCAGT-3′
	Reverse: 5′-ATTACCGCGGCTGCTGGC-3′

*Firmicutes*	Forward: 5′-GCGTGAGTGAAGAAGT-3′
	Reverse: 5′-CTACGCTCCCTTTACAC-3′

*Bacteroidetes*	Forward: 5′-ACGCTAGCTACAGGCTTAACA-3′
	Reverse: 5′-ACGCTACTTGGCTGGTTCA-3′

*Lactobacillus* sp.	Forward:5′-CACCGCTACACATGGAG-3′
	Reverse: 5′-AGCAGTAGGGAATCTTCCA-3′

*Bifidobacterium* sp.	Forward:5′-TCGCGTCYGGTGTGAAAG-3′
	Reverse: 5′-CCACATCCAGCRTCCAC-3′

**Table 3:** The levels of TG, TC, LDL-C, and HDL-C in serum of mice.

Group	Normal	Model	AX	HFY06	Ax+HFY06
TG (mmol/L)	0.52 ± 0.06^a^	0.96 ± 0.06^b^	0.57 ± 0.08^a^	0.56 ± 0.15^a^	0.54 ± 0.07^a^
TC (mmol/L)	2.29 ± 0.31^a^	3.77 ± 0.34^c^	2.74 ± 0.39^a^	2.83 ± 0.41^a^	2.95 ± 0.05^a^
LDL-C (mmol/L)	0.31 ± 0.10^a^	0.76 ± 0.10^c^	0.67 ± 0.10^c^	0.47 ± 0.11^b^	0.45 ± 0.10^b^
HDL-C (mmol/L)	0.33 ± 0.04^b^	0.23 ± 0.04^a^	0.29 ± 0.07^ab^	0.26 ± 0.03^a^	0.30 ± 0.04^ab^

Data are means ± SD (*n* =10 per group). ^a–c^Mean values with different letters in the same column are significantly different (*p* < 0.05) according to Duncan's multiple range test. AX: high-fat diet + AX (200 mg/kg); HFY06: high fat diet + *L. fermentum* HFY06 (1.0 × 10^10^ CFU/kg); AX+HFY06: high-fat diet + AX (200 mg/kg) + *L. fermentum* HFY06 (1.0 × 10^10^ CFU/kg).

**Table 4:** The levels of CAT, SOD, NO, and MDA in serum of mice.

Group	Normal	Model	AX	HFY06	Ax+HFY06
CAT (U/mL)	80.92 ± 3.04^a^	51.82 ± 3.77^b^	53.91 ± 1.97^a^	53.96 ± 5.95^a^	54.61 ± 3.50^a^
SOD (U/mL)	45.20 ± 2.58^a^	44.58 ± 2.47^a^	45.86 ± 4.15^a^	45.73 ± 5.88^a^	47.90 ± 2.02^a^
NO (*μ*mol/L)	7.32 ± 2.56^a^	14.43 ± 1.85^b^	14.01 ± 1.35^b^	9.80 ± 2.11^a^	8.64 ± 3.56^a^
MDA (nmol/mL)	1.10 ± 0.25^a^	3.22 ± 0.52^d^	2.17 ± 0.97^c^	1.99 ± 0.55^bc^	1.21 ± 0.16^ab^

Data are means ± SD (*n* =10 per group). ^a–b^Mean values with different letters in the same column are significantly different (*p* < 0.05) according to Duncan's multiple range test. AX: high-fat diet + AX (200 mg/kg); HFY06: high-fat diet + *L. fermentum* HFY06 (1.0 × 10^10^ CFU/kg); AX+HFY06: high-fat diet + AX (200 mg/kg) + *L. fermentum* HFY06 (1.0 × 10^10^ CFU/kg).

**Table 5:** The levels of INF-*γ*, IL-1*β*, IL-6, TFN-*α*, and IL-10 in serum of mice.

Group	Normal	Model	AX	HFY06	Ax+HFY06
IFN-*γ* (pg/mL)	840.44 ± 28.38^b^	904.98 ± 31.08^c^	791.53 ± 34.08^a^	788.75 ± 42.85^a^	795.03 ± 25.95^a^
IL-1*β* (pg/mL)	29.21 ± 1.03^ab^	32.02 ± 1.06^c^	28.24 ± 1.28^a^	30.51 ± 1.53^bc^	30.58 ± 1.15^bc^
IL-6 (pg/mL)	38.02 ± 1.19^a^	42.16 ± 2.37^b^	38.62 ± 1.16^a^	38.81 ± 2.77^a^	38.93 ± 2.02^a^
TNF-*α* (pg/mL)	661.26 ± 64.40^a^	767.20 ± 31.21^c^	707.30 ± 18.60^b^	683.91 ± 44.83^ab^	649.75 ± 35.38^a^
IL-10 (pg/mL)	503.23 ± 8.86^b^	463.87 ± 19.90^a^	467.13 ± 28.70^a^	485.86 ± 8.01^ab^	485.30 ± 9.07^ab^

Data are means ± SD (*n* =10 per group). ^a–c^Mean values with different letters in the same column are significantly different (*p* < 0.05) according to Duncan's multiple range test. AX: high-fat diet + AX (200 mg/kg); HFY06: high-fat diet + *L. fermentum* HFY06 (1.0 × 10^10^ CFU/kg); AX+HFY06: high-fat diet + AX (200 mg/kg) + *L. fermentum* HFY06 (1.0 × 10^10^ CFU/kg).

## Data Availability

The datasets generated for this study are available upon request to the corresponding author.

## Abbreviations

L. fermentum HFY06: Lactobacillus fermentum HFY06
LFD: Low-fat diet
HFD: High-fat diet
AX: Arabinoxylan
AMPK: AMP-activated protein kinase
CAT: Catalase
MDA: Malondialdehyde
SOD: Superoxide dismutase
H&E: Hematoxylin and eosin
NO: Nitric oxide
CPT-1: Carnitine palmitoyl transferase 1
PPAR-*α*: Peroxisome proliferators-activated receptors *α*
CYP7A1: Cholesterol 7-alpha hydroxylase
HSL: Hormone-sensitive lipase
ACC: Acetyl-CoA carboxylase
C/EBP*α*: CCAAT/enhancer-binding protein alpha
LPL: Lipoprotein lipase
ZO-1: Zonula occludens-1
TG: Triglycerides
TC: Total cholesterol
LDL-C: Low-density lipoprotein cholesterol
HDL-C: High-density lipoprotein cholesterol
IL-6: Interleukin-6
IL-10: Interleukin-10
IL-1*β*: Interleukin-1*β*
IFN-*γ*: Interferon-gamma
TNF-*α*: Tumor necrosis factor alpha
ALT: Alanine aminotransferase
AST: Aspartate aminotransferase.

## Supplementary Materials

Supplementary Materials Figure S1 and S2 are the melting curves of corresponding mRNA expression.
