# Supplementary material for: Effect of Lactobacillus fermentum HFY06 Combined with Arabinoxylan on Reducing Lipid Accumulation in Mice Fed with High-Fat Diet
Source: Oxid Med Cell Longev. 2022 Apr 6;2022:1068845. doi: 10.1155/2022/1068845 (PMC9007687; doi:10.1155/2022/1068845)
Supplement: Supplementary Materials — Figure S1 and S2 are the melting curves of corresponding mRNA expression. [file 1068845.f1.zip › Figure supplement 1.pdf]

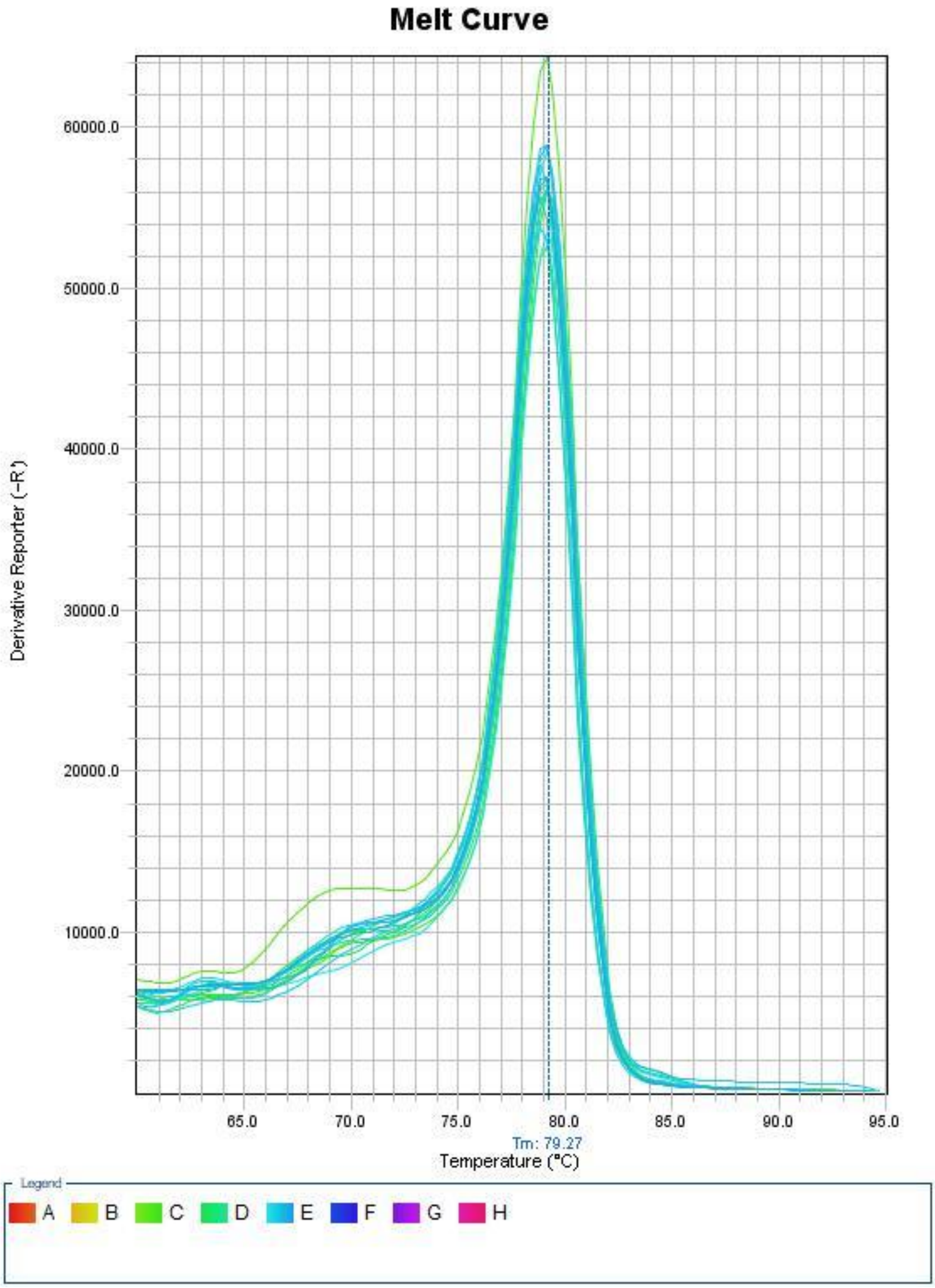

Melt Curve acc FAT

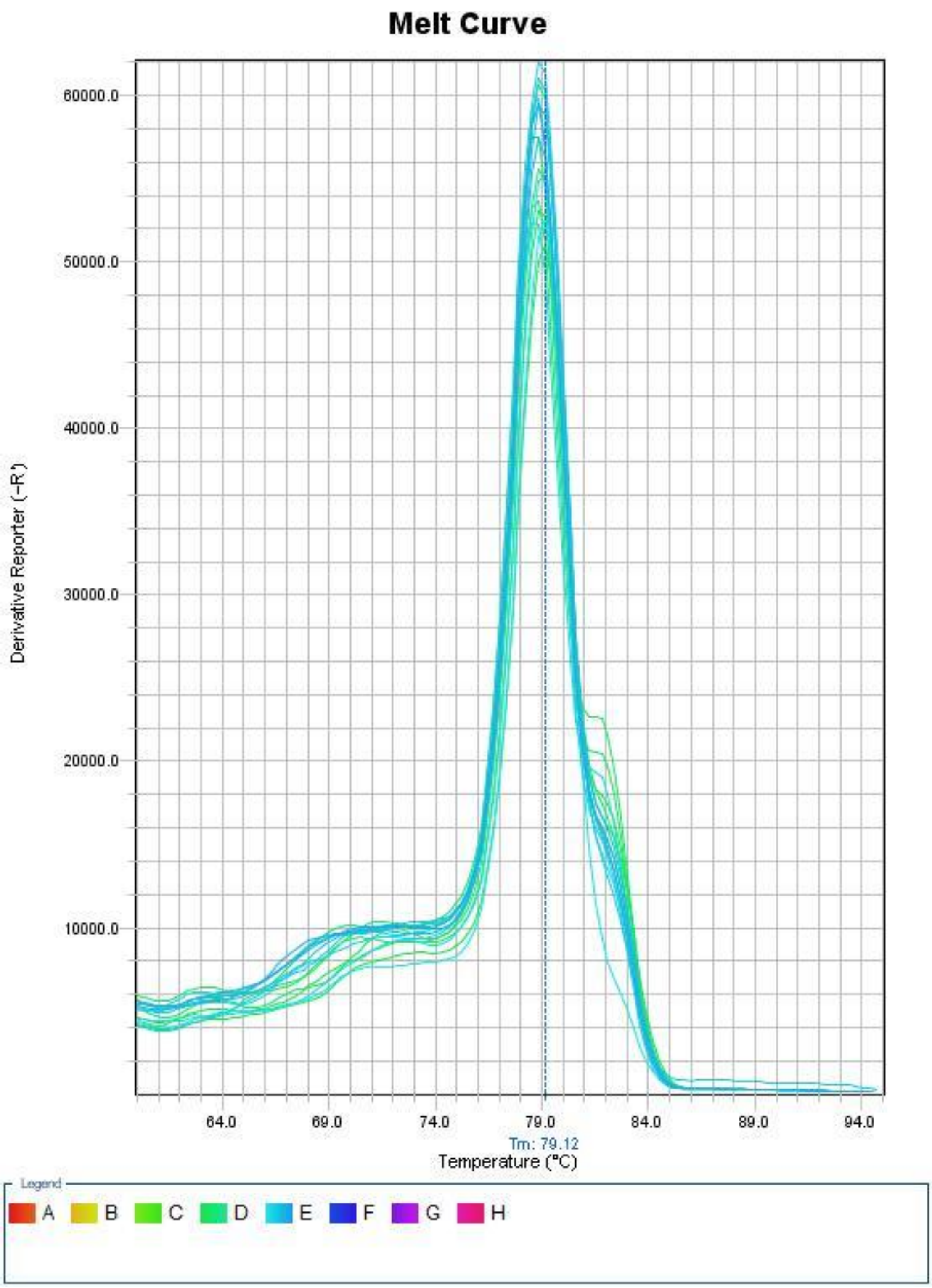

Melt Curve AMPK FAT

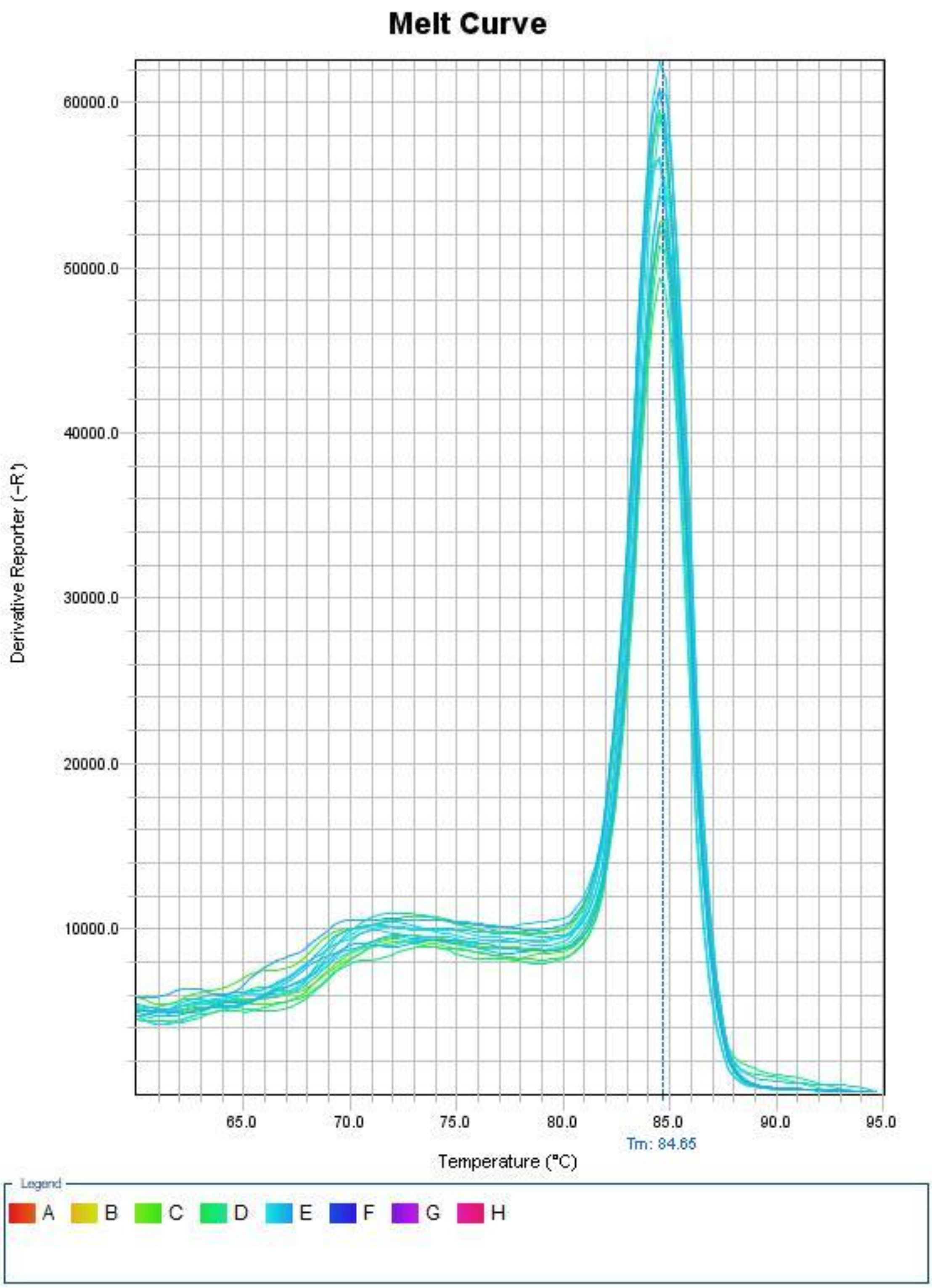

Melt Curve CEBP FAT

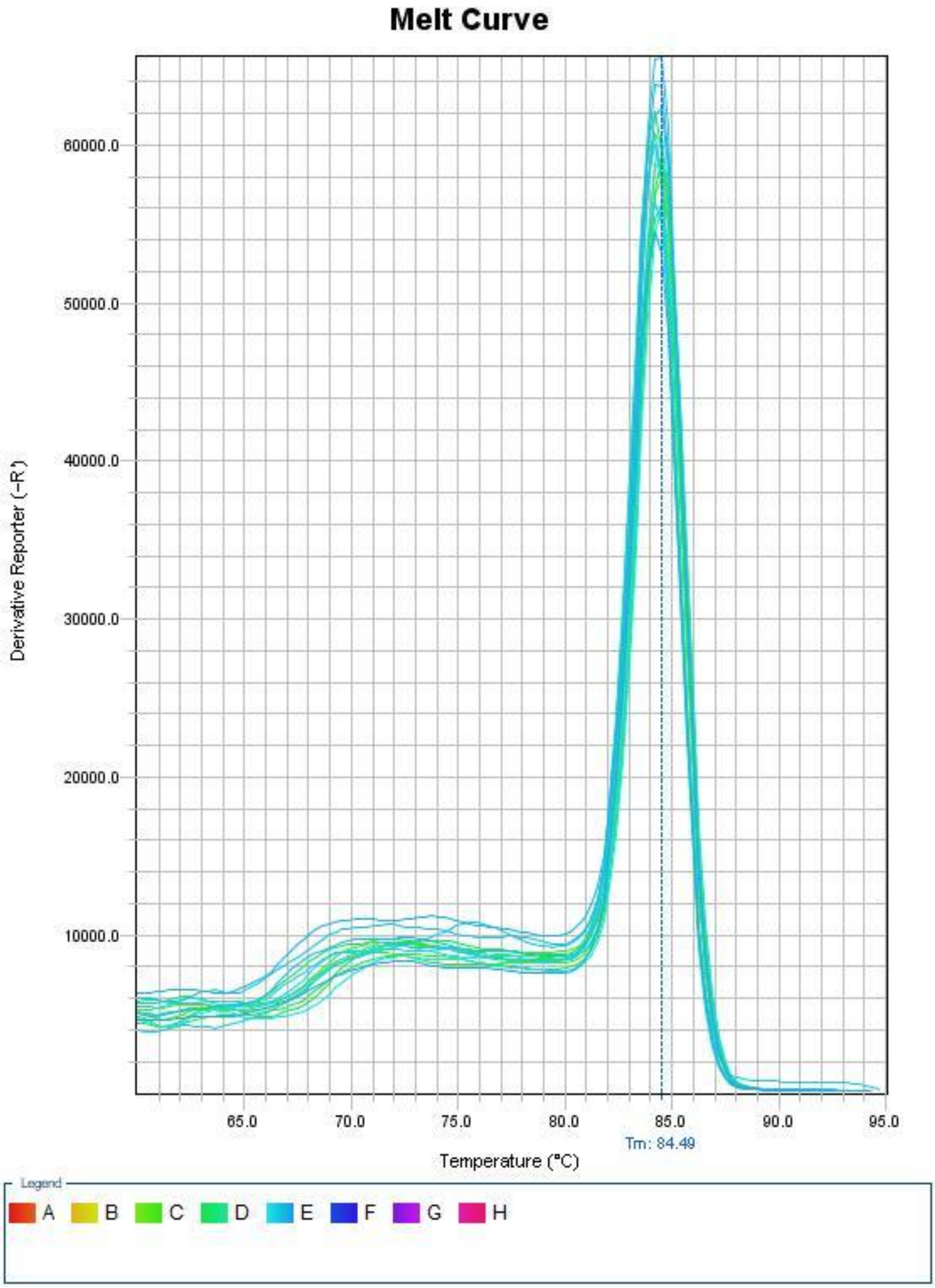

Melt Curve CPT-1 FAT

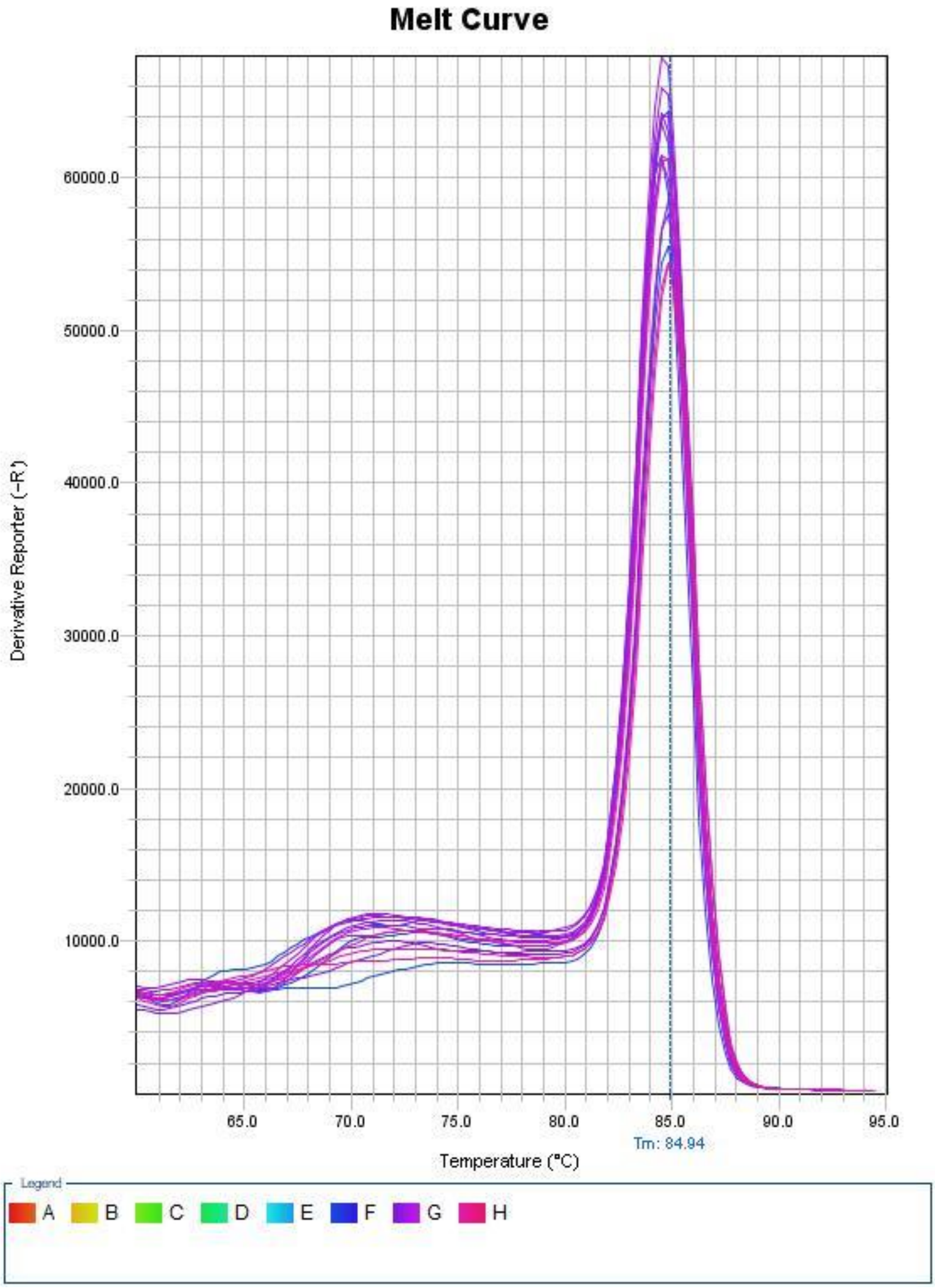

Melt Curve hsl FAT

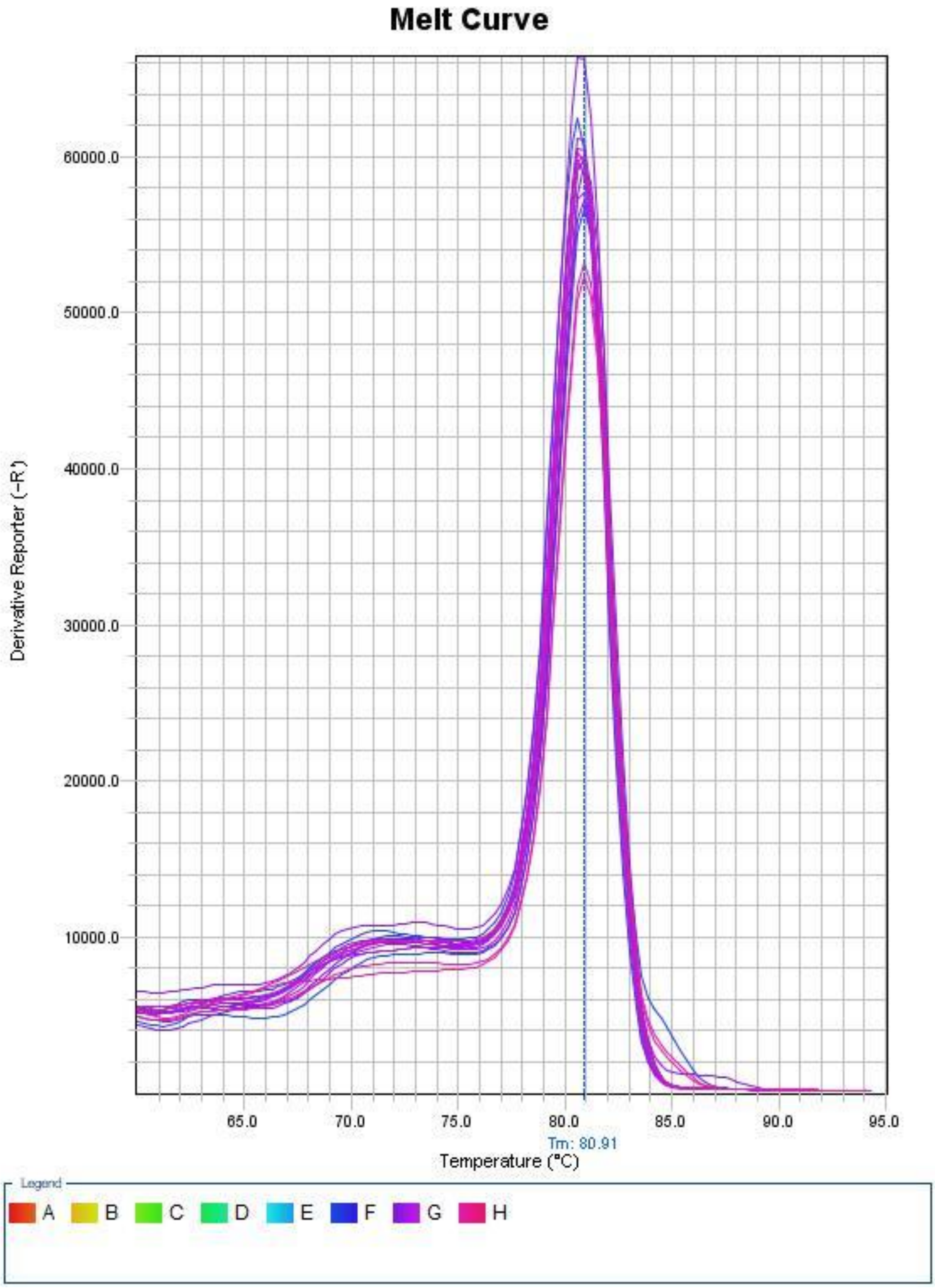

Melt Curve LPL FAT

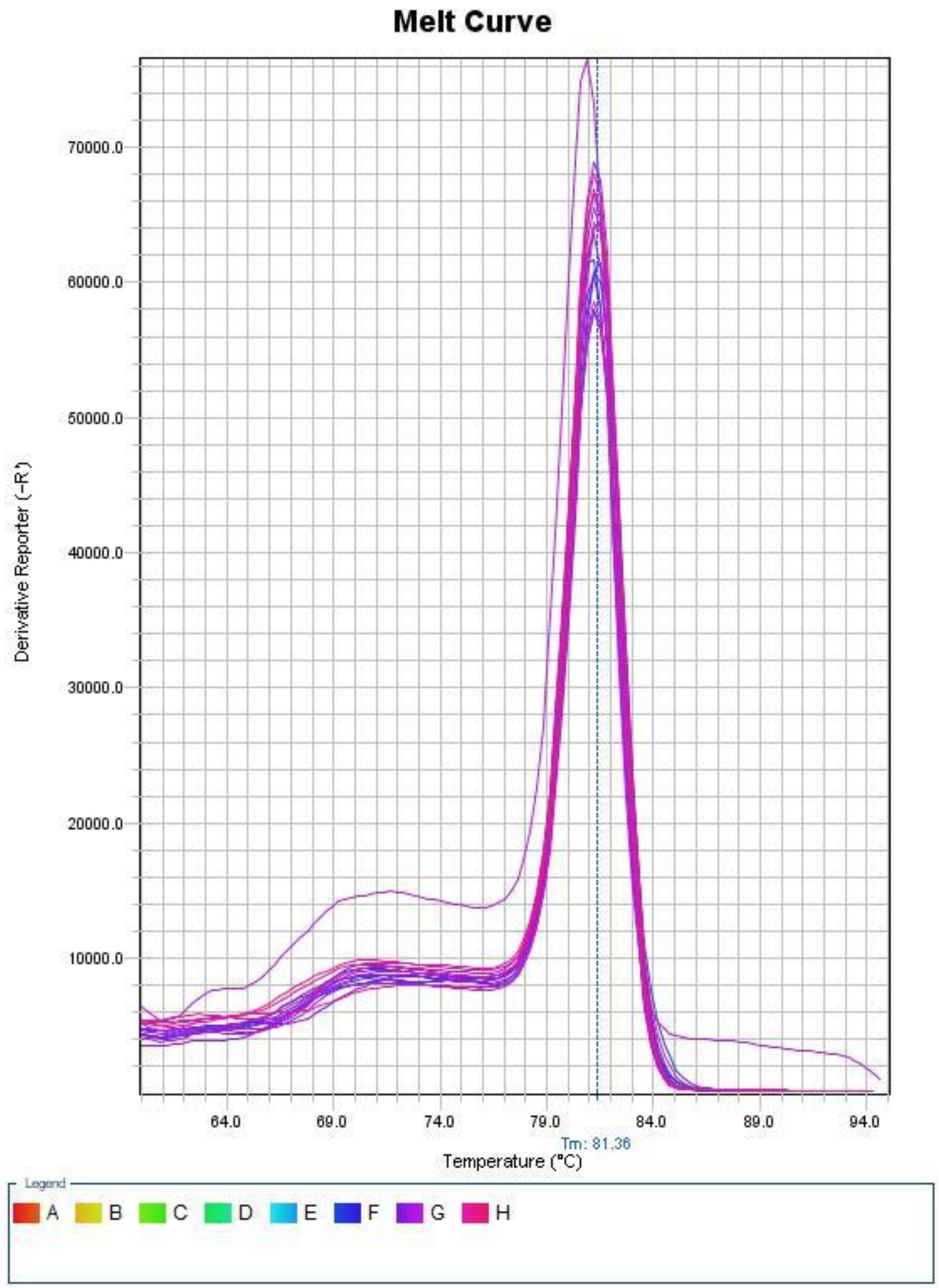

Melt Curve PPAR-A FAT
