# Supplementary material for: Effect of Lactobacillus fermentum HFY06 Combined with Arabinoxylan on Reducing Lipid Accumulation in Mice Fed with High-Fat Diet
Source: Oxid Med Cell Longev. 2022 Apr 6;2022:1068845. doi: 10.1155/2022/1068845 (PMC9007687; doi:10.1155/2022/1068845)
Supplement: Supplementary Materials — Figure S1 and S2 are the melting curves of corresponding mRNA expression. [file 1068845.f1.zip › Figure supplement 2.pdf]

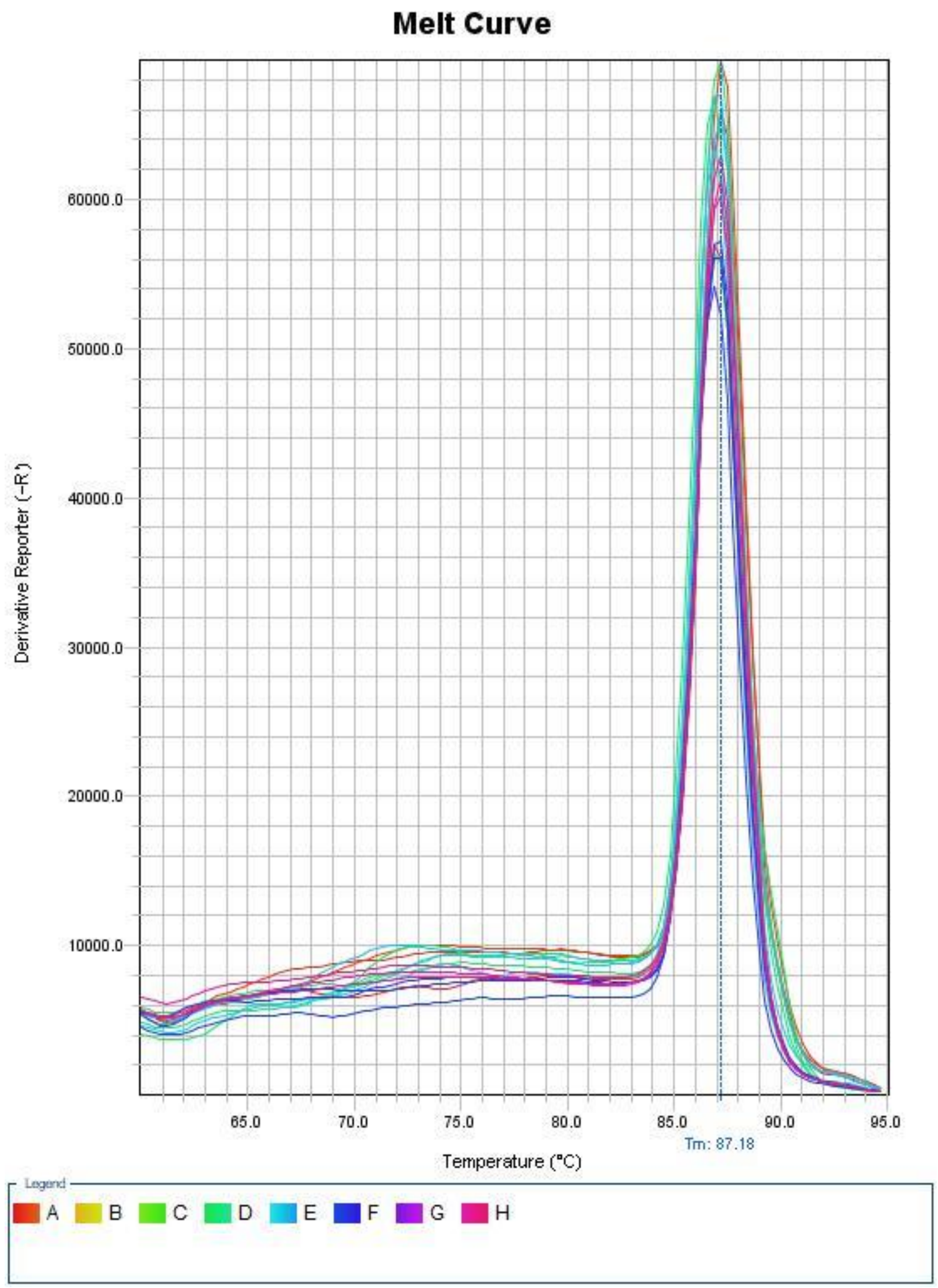

Melt Curve   Bifidobacterium sp

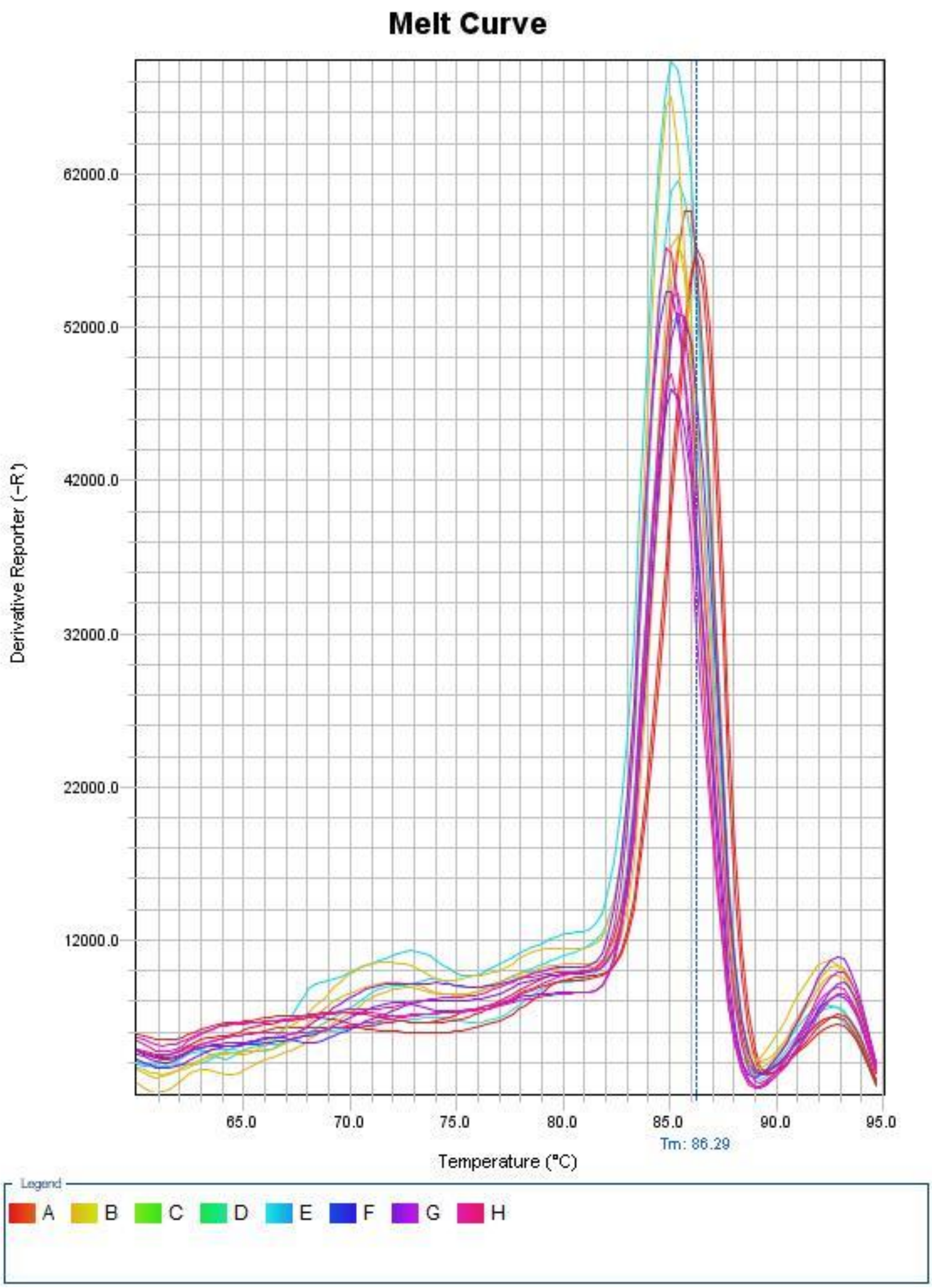

Melt Curve Lactobacillus sp

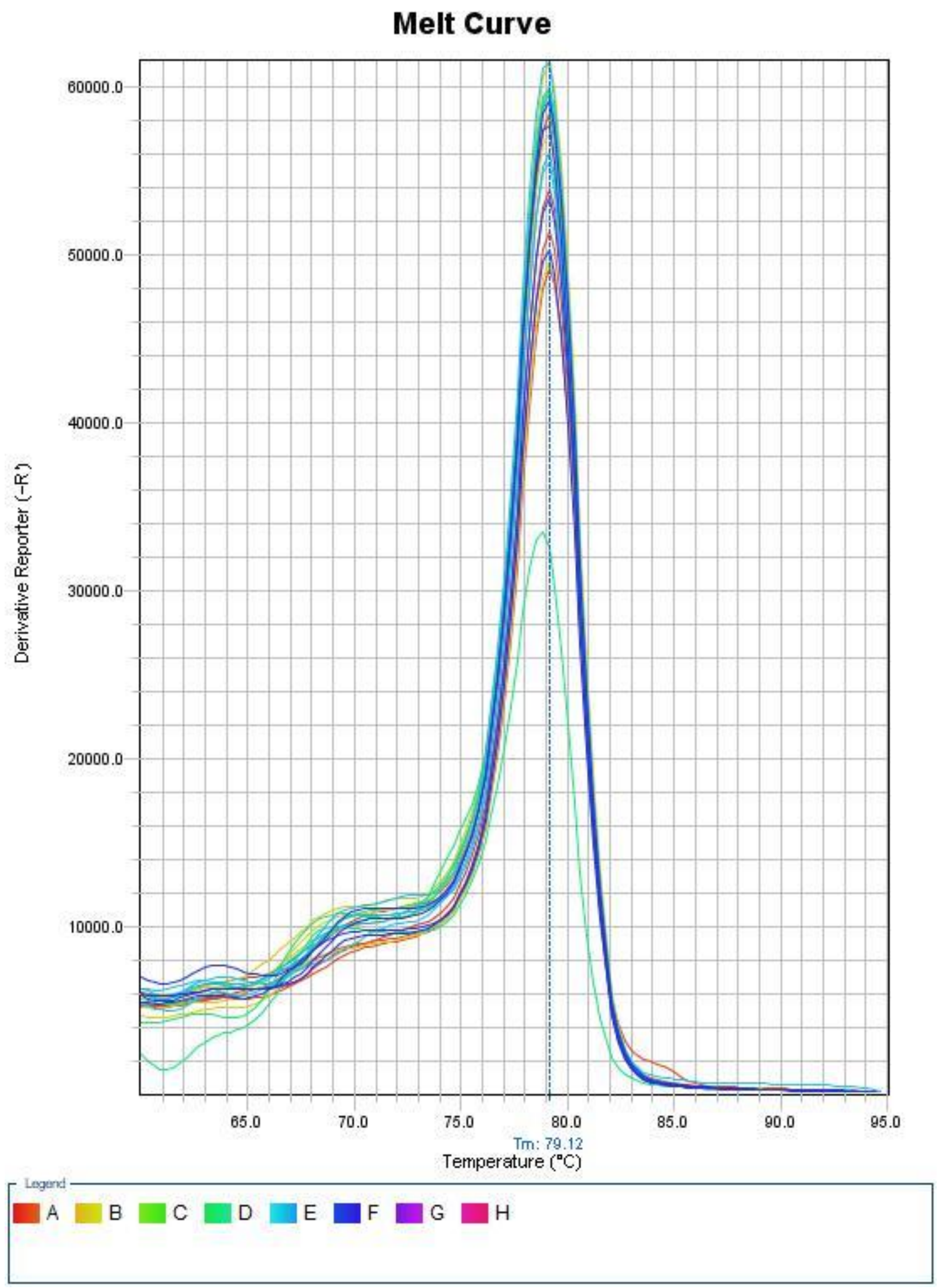

Melt Curve acc

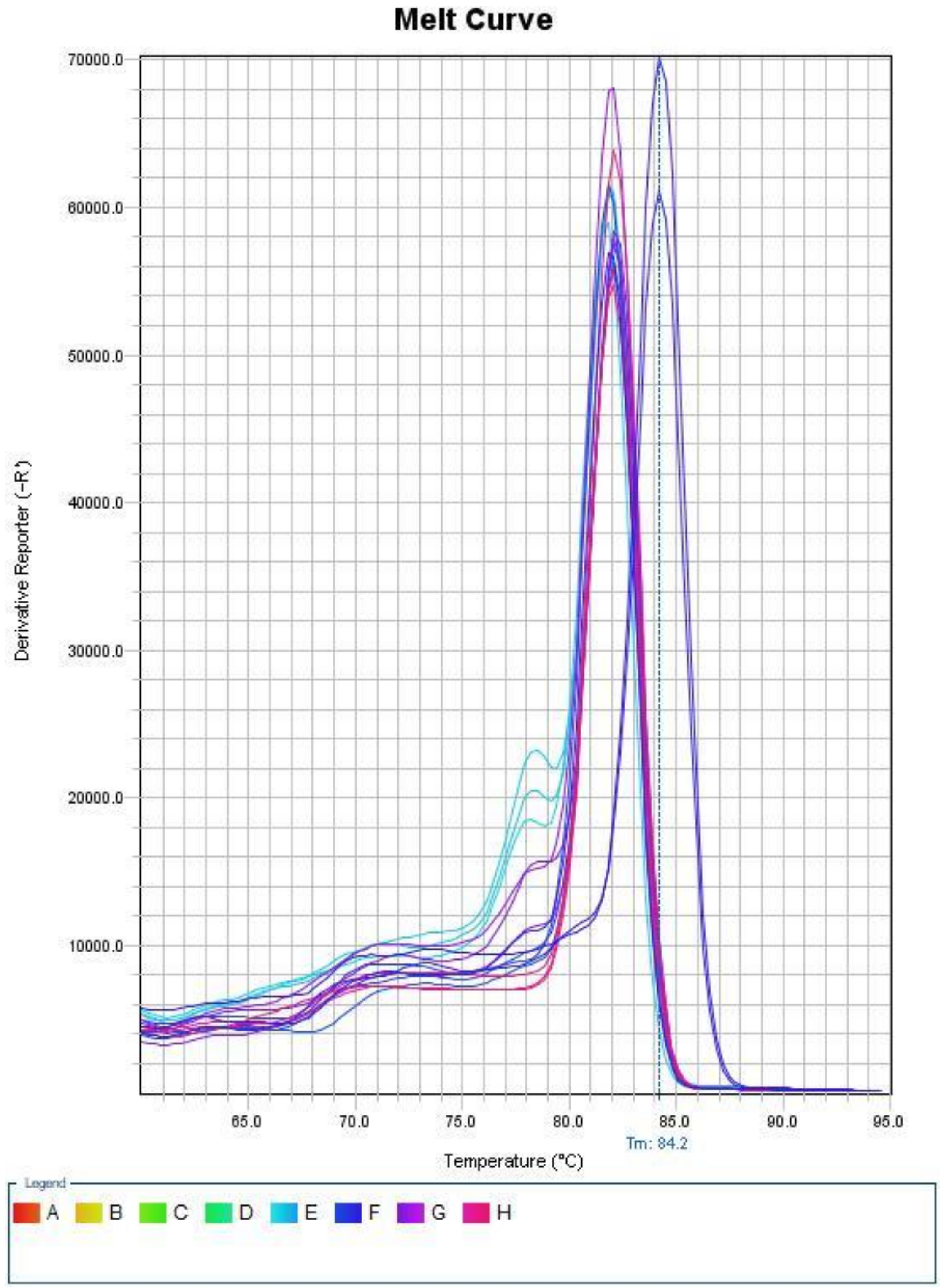

Melt Curve ampk

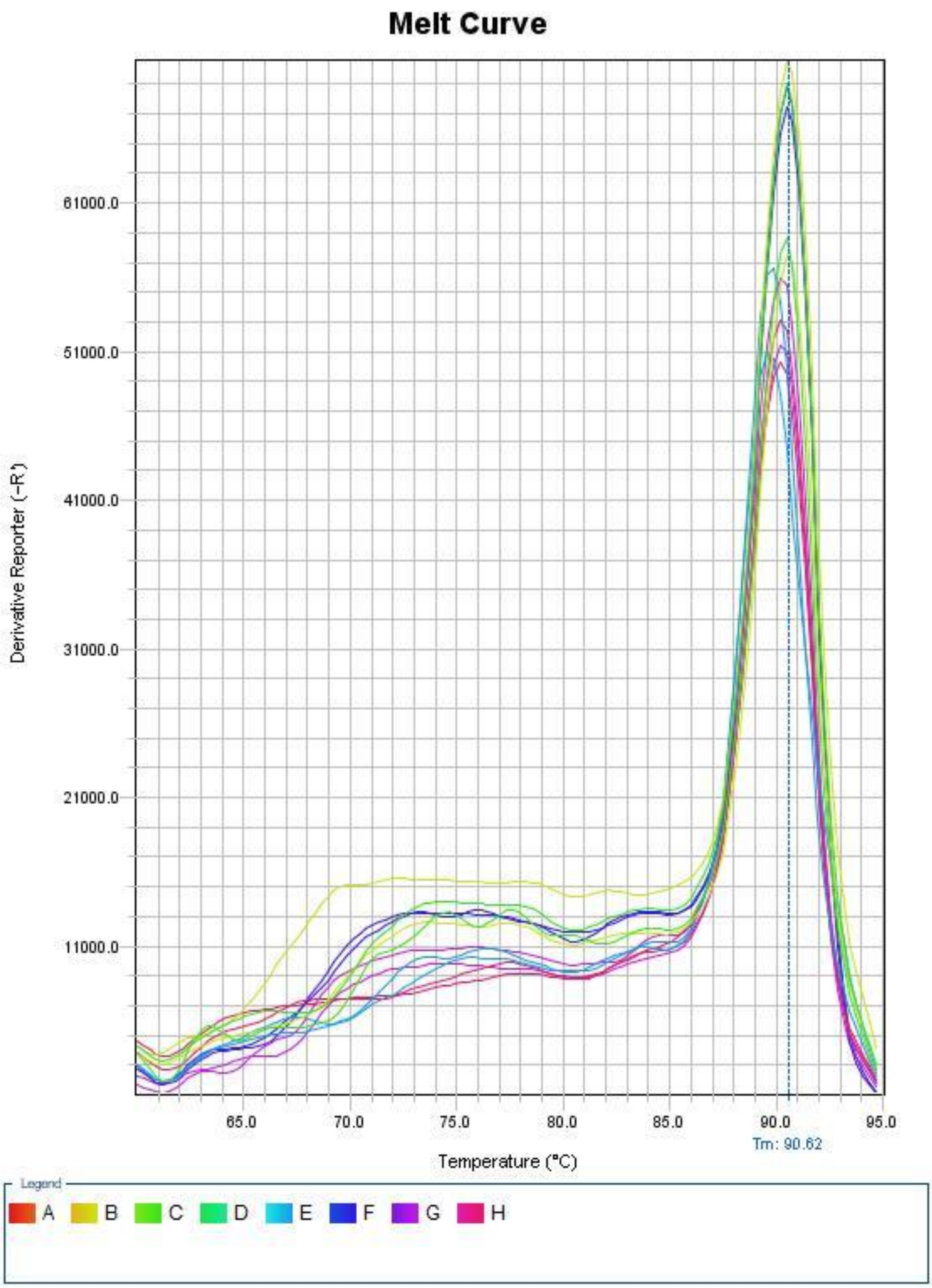

Melt Curve Bacteroides

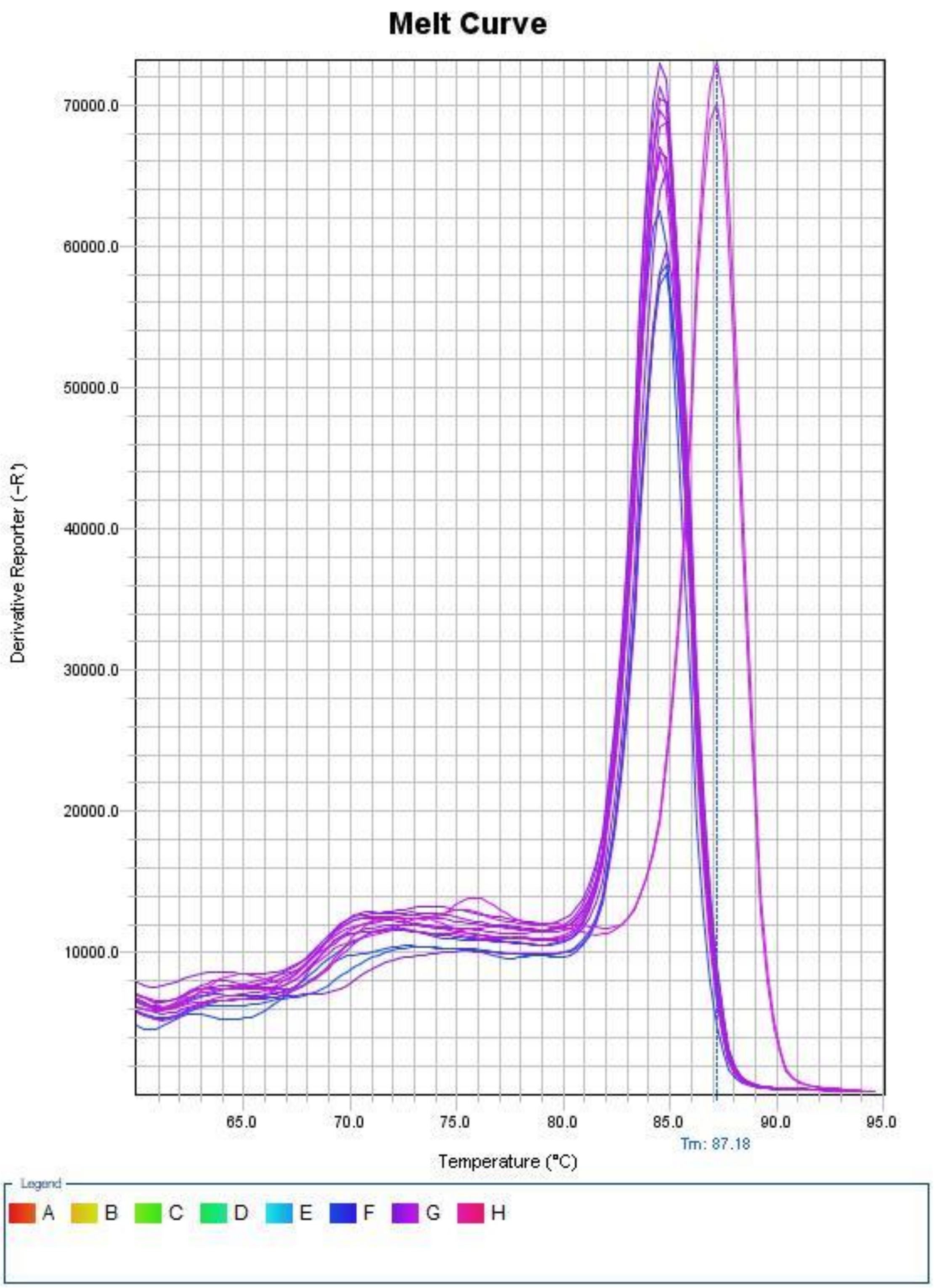

Melt Curve cebp

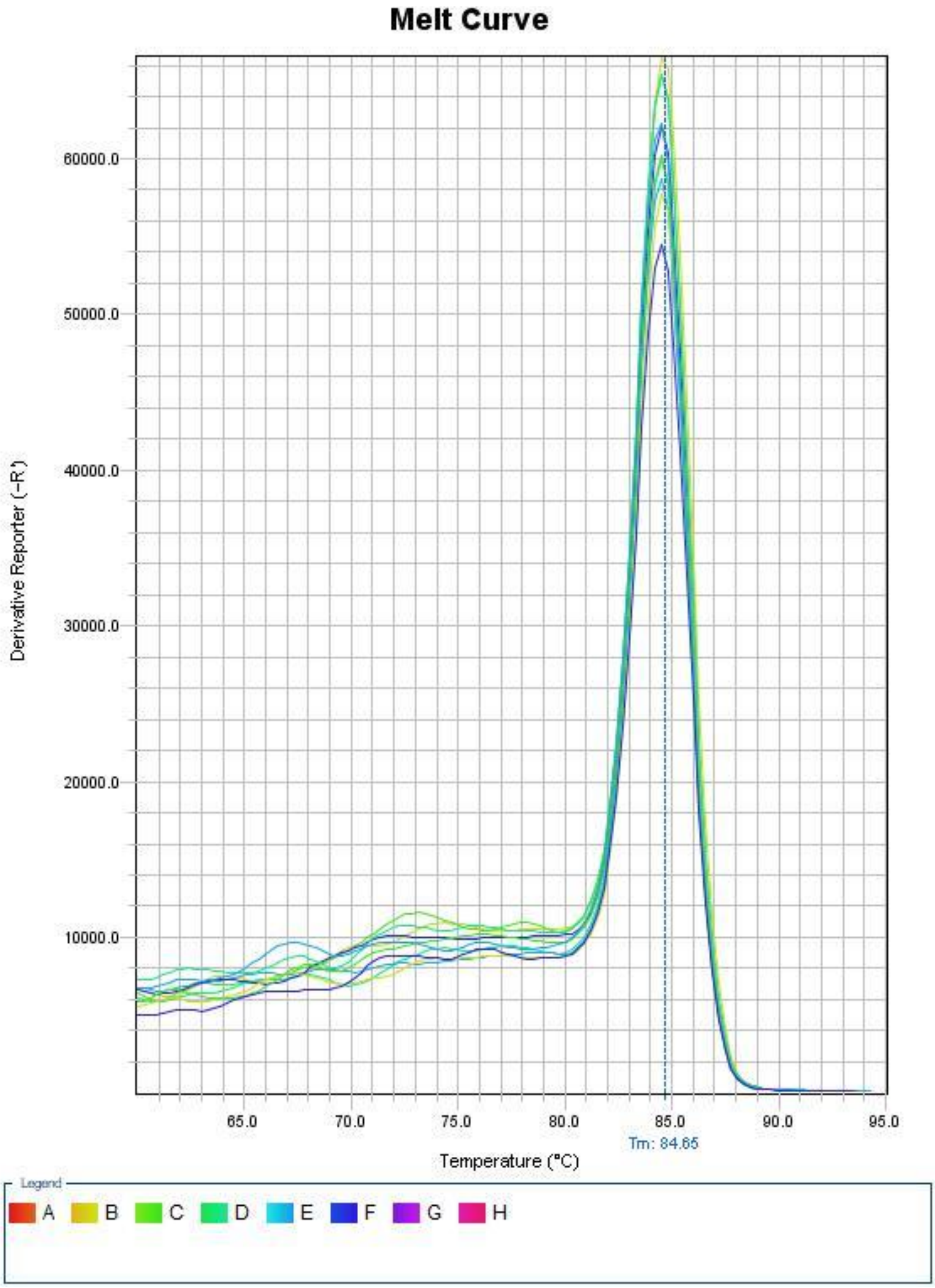

Melt Curve claudin-1

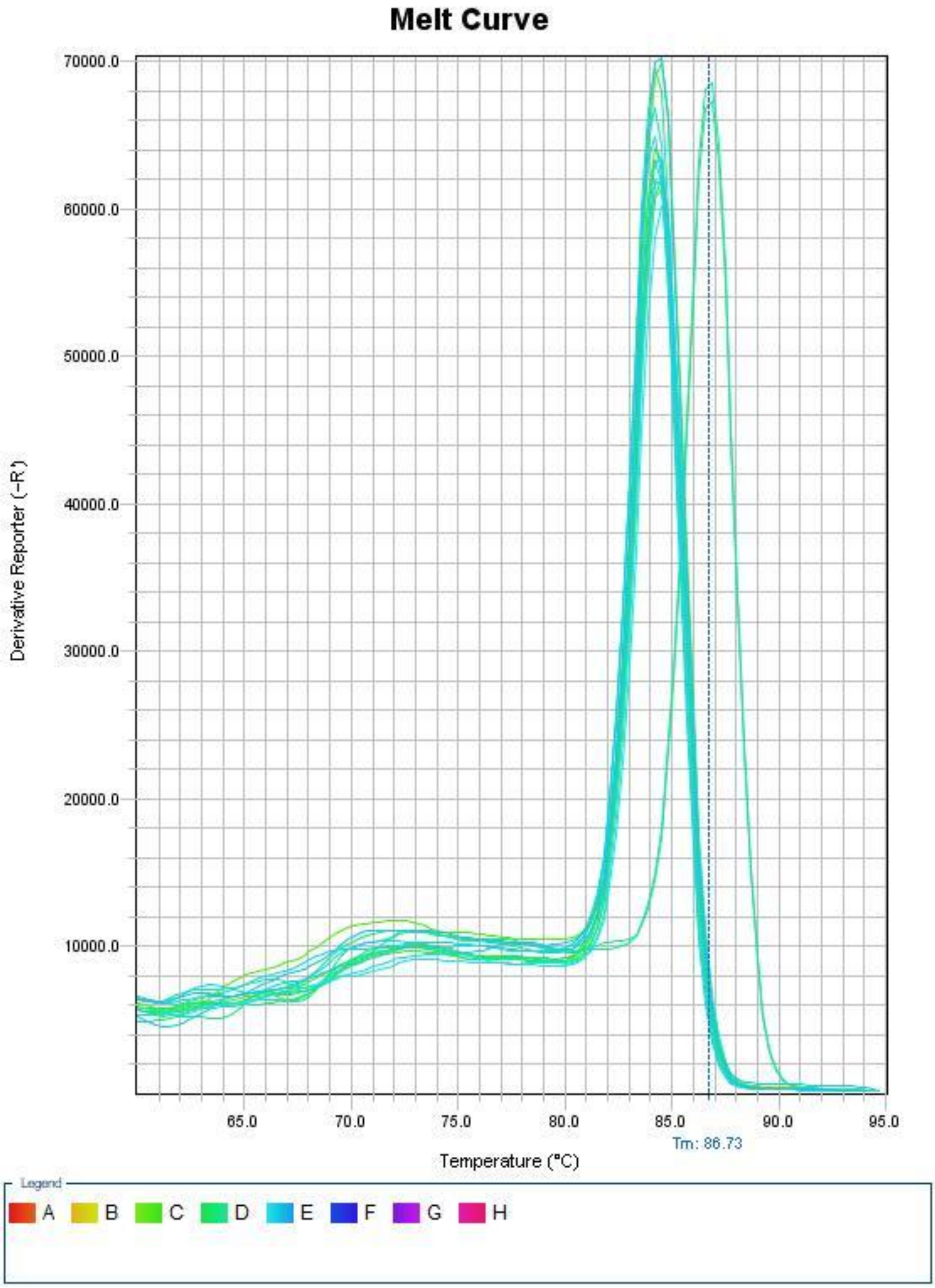

Melt Curve cpt1

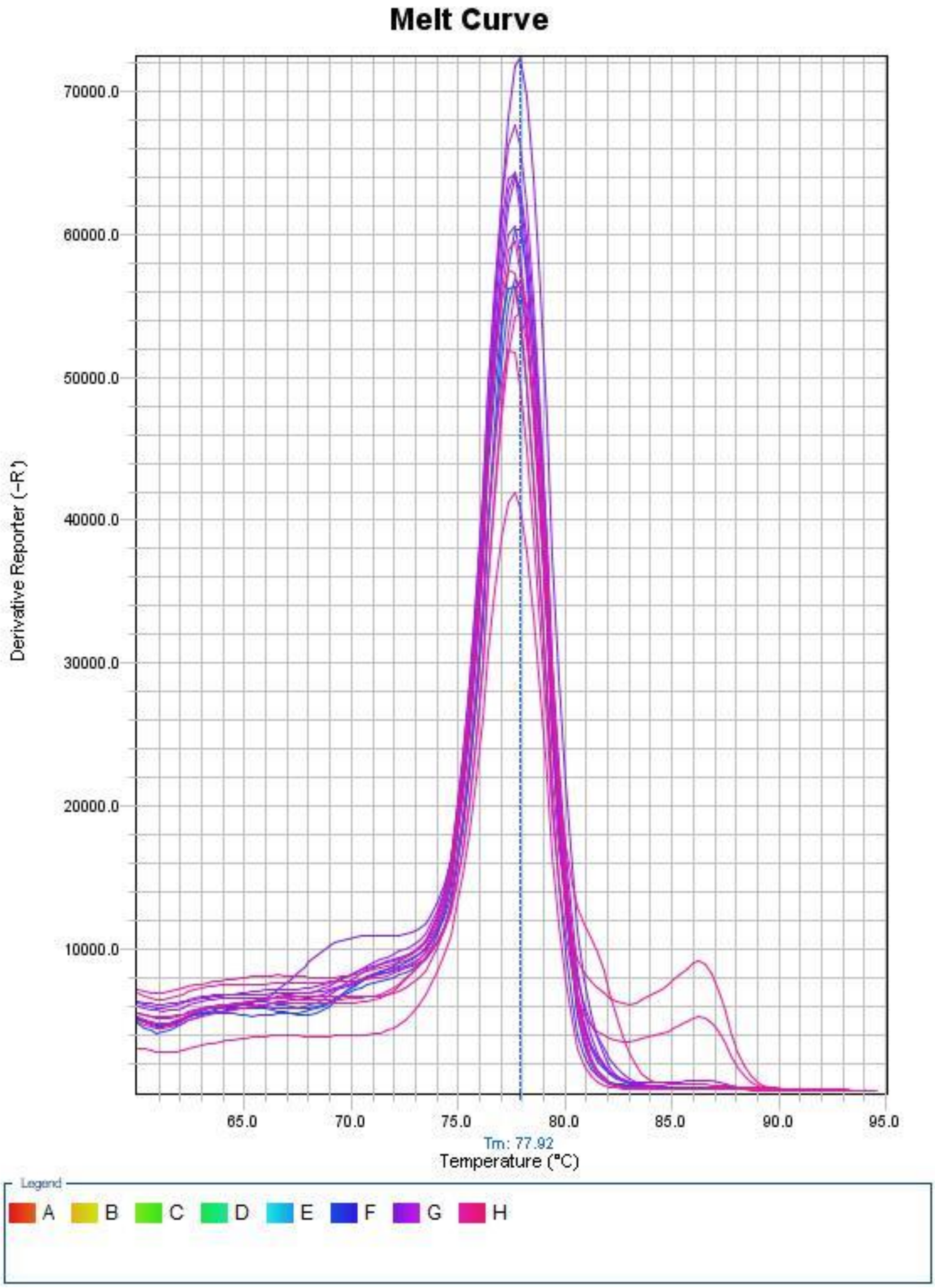

Melt Curve cyp7a1

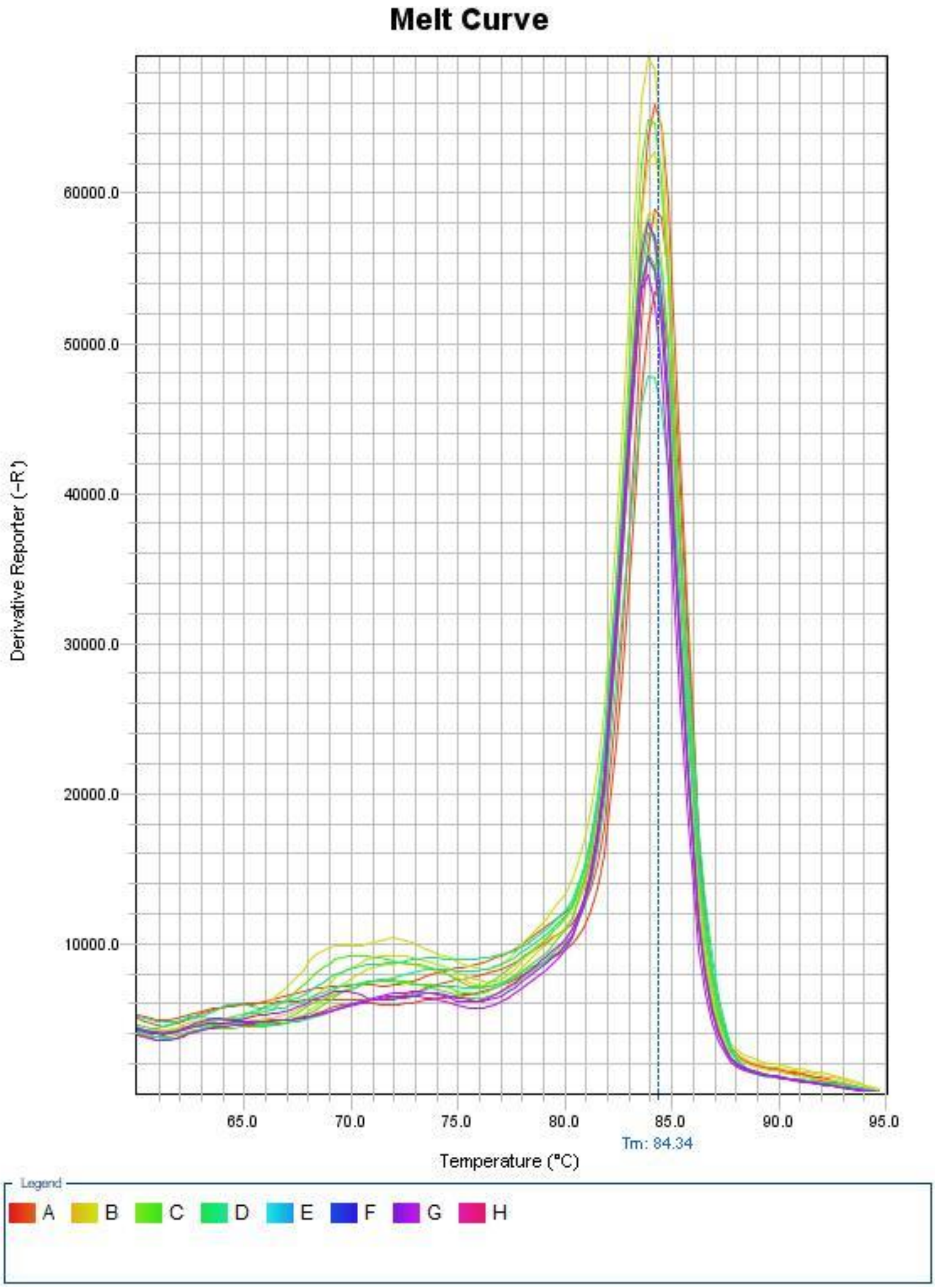

Melt Curve firmicutes

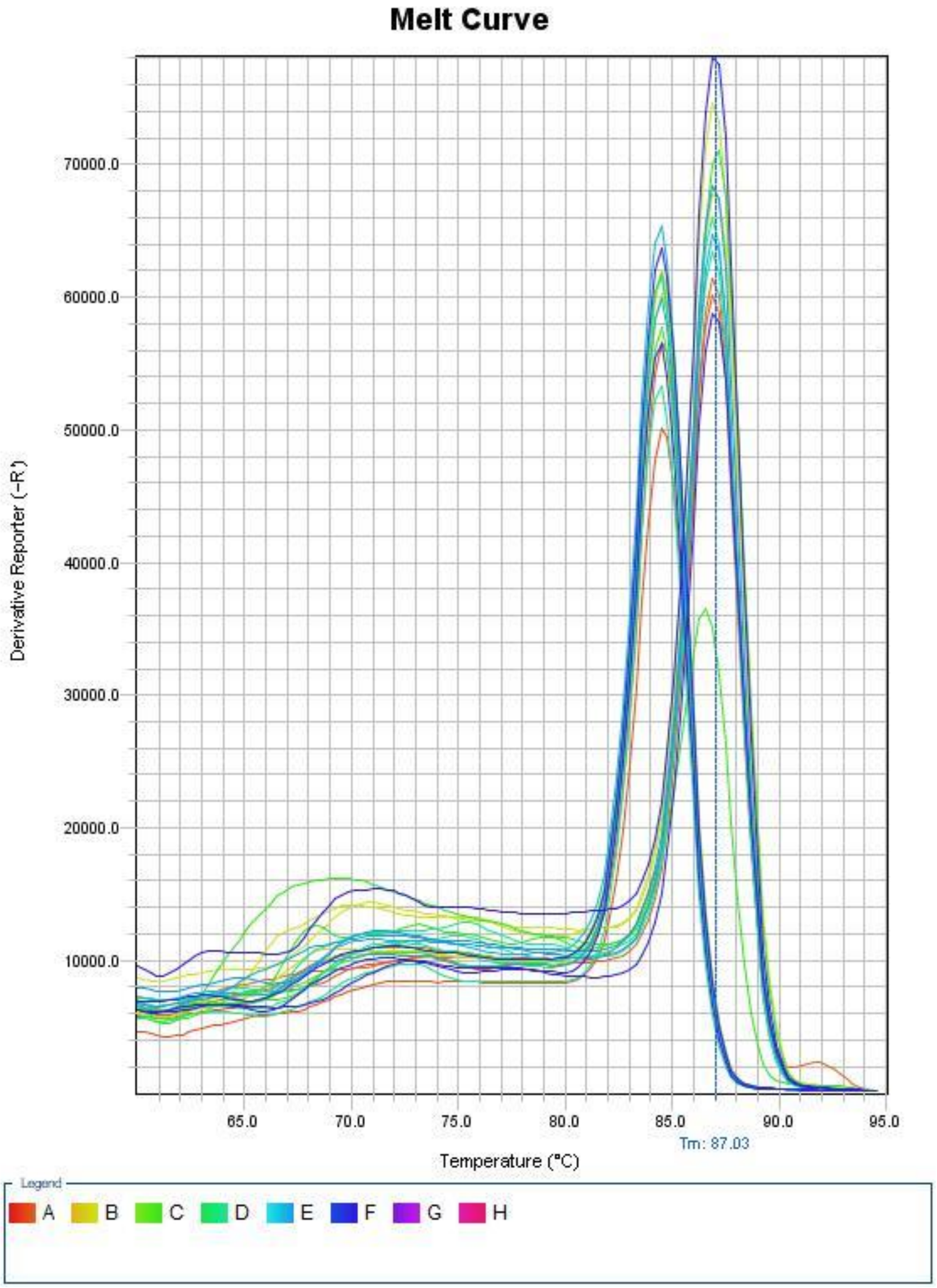

Melt Curve hsl

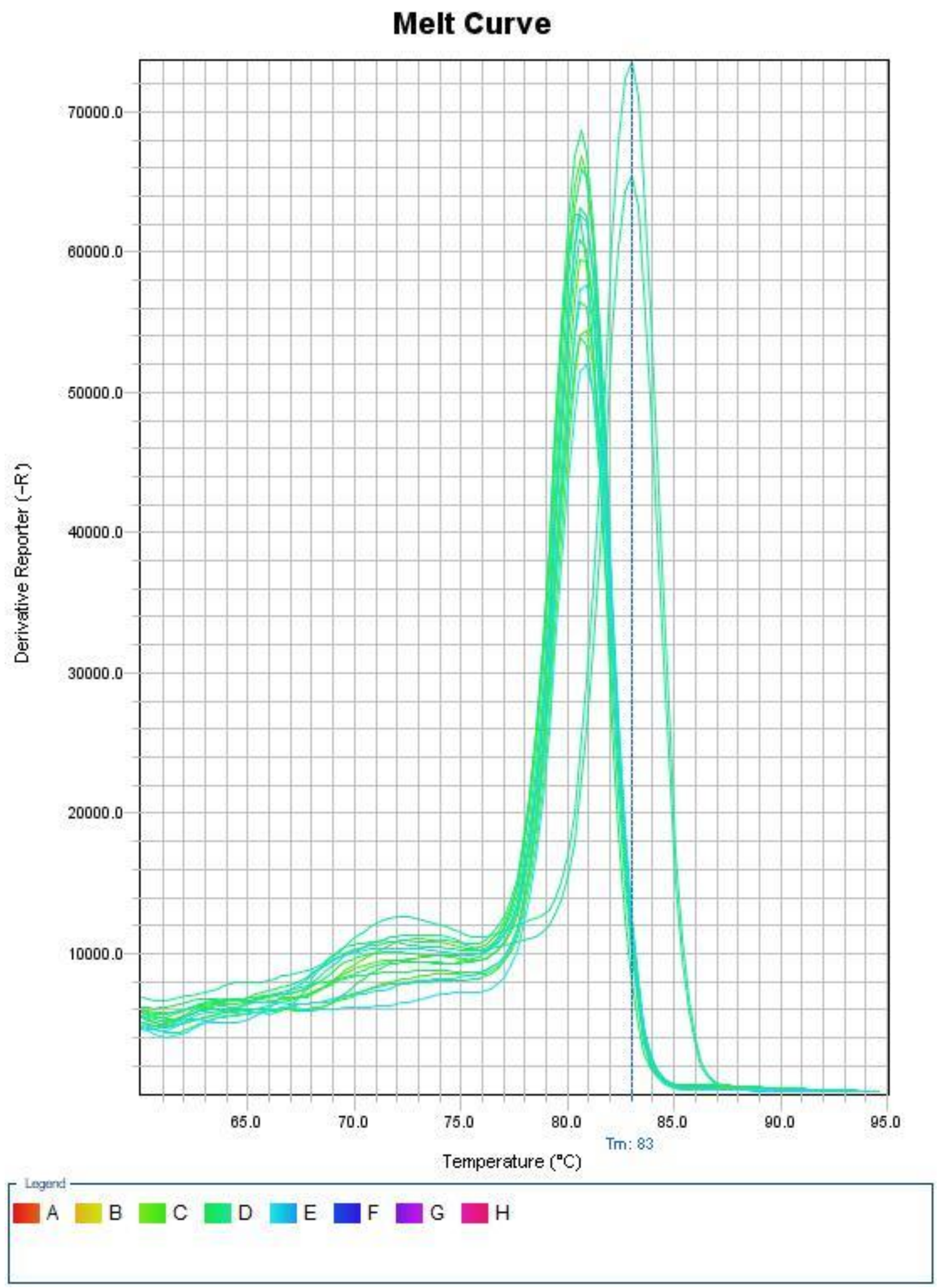

Melt Curve lpl

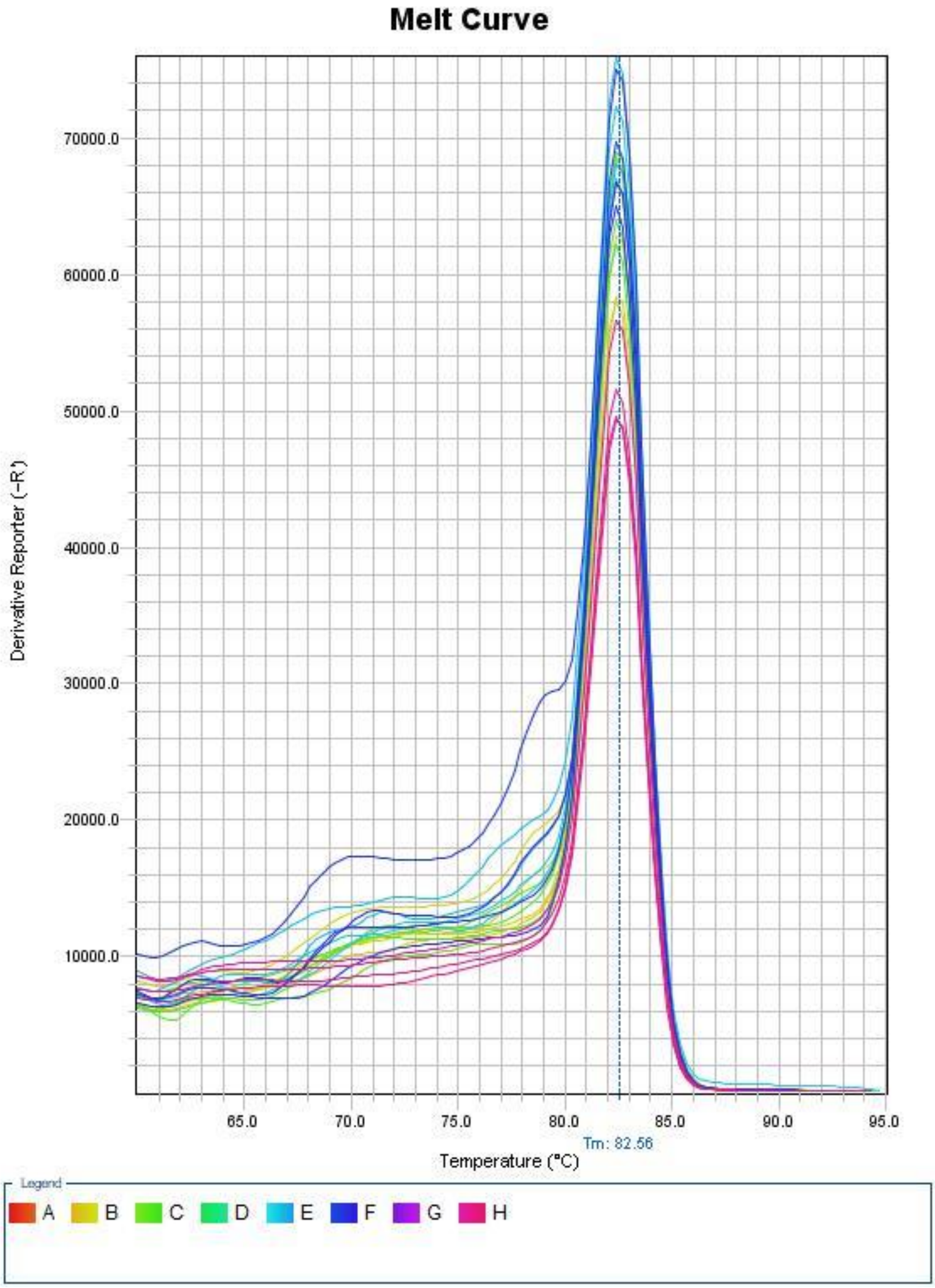

Melt Curve occluding

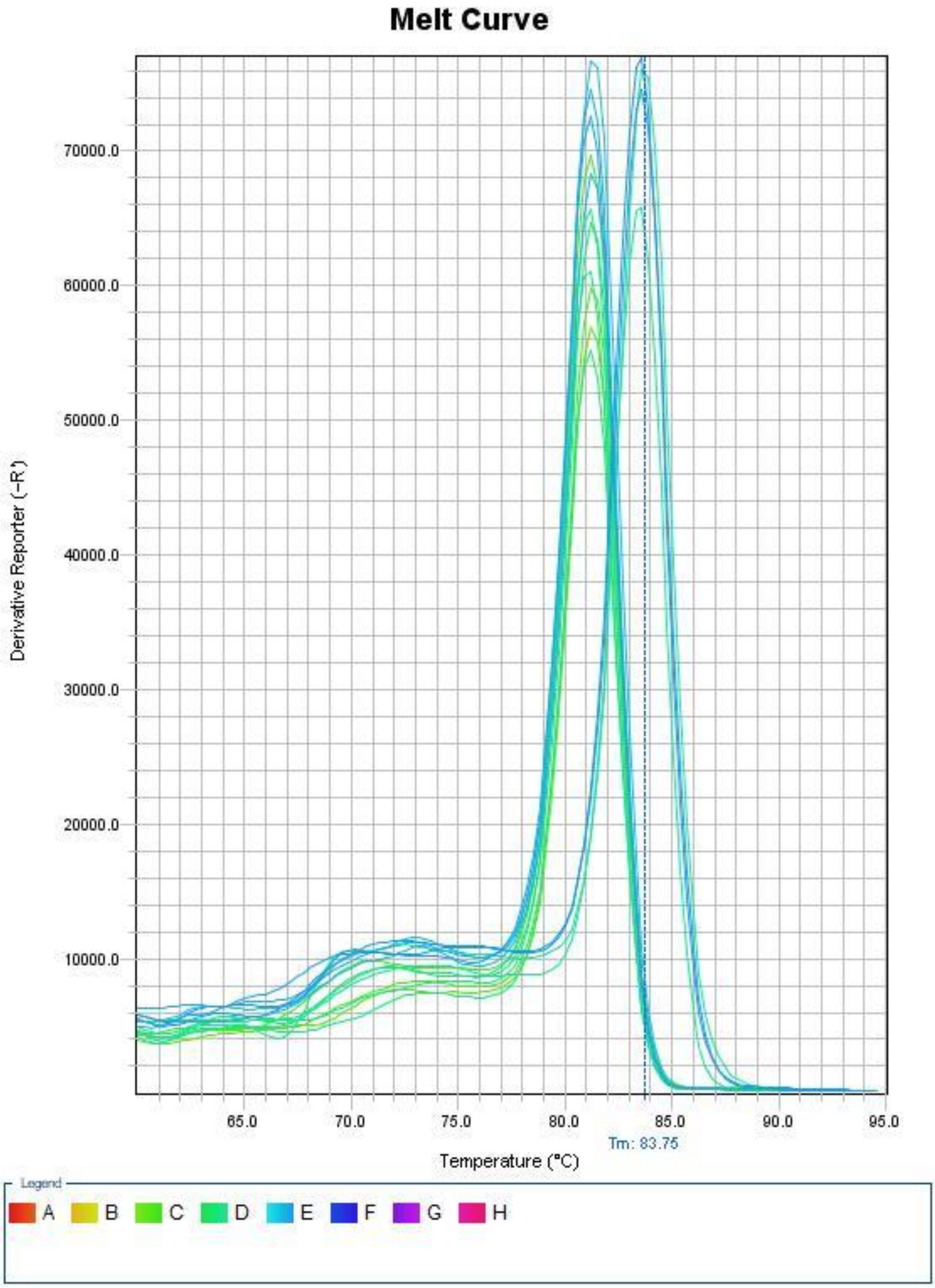

Melt Curve ppar-a

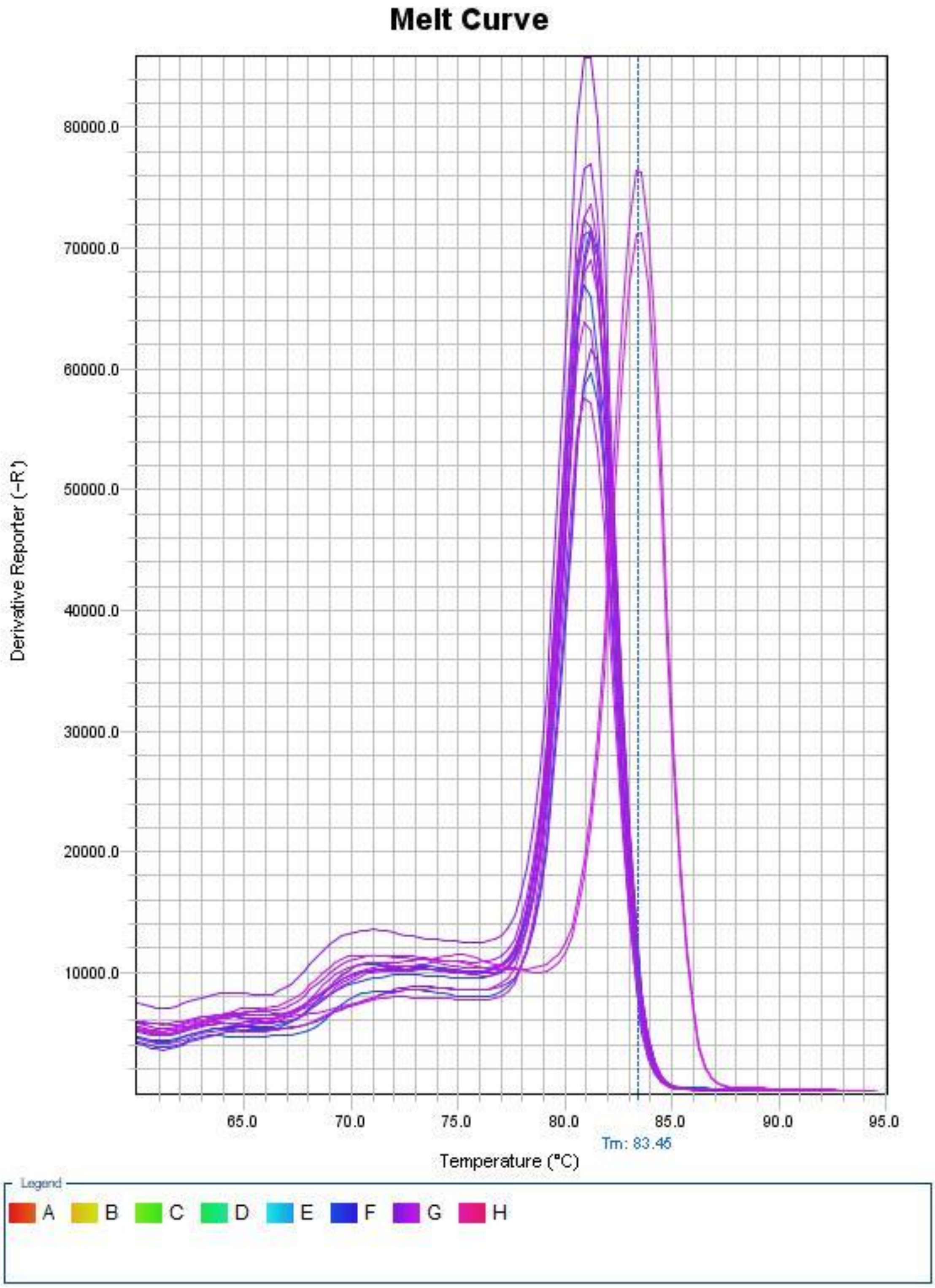

Melt Curve ppar-r

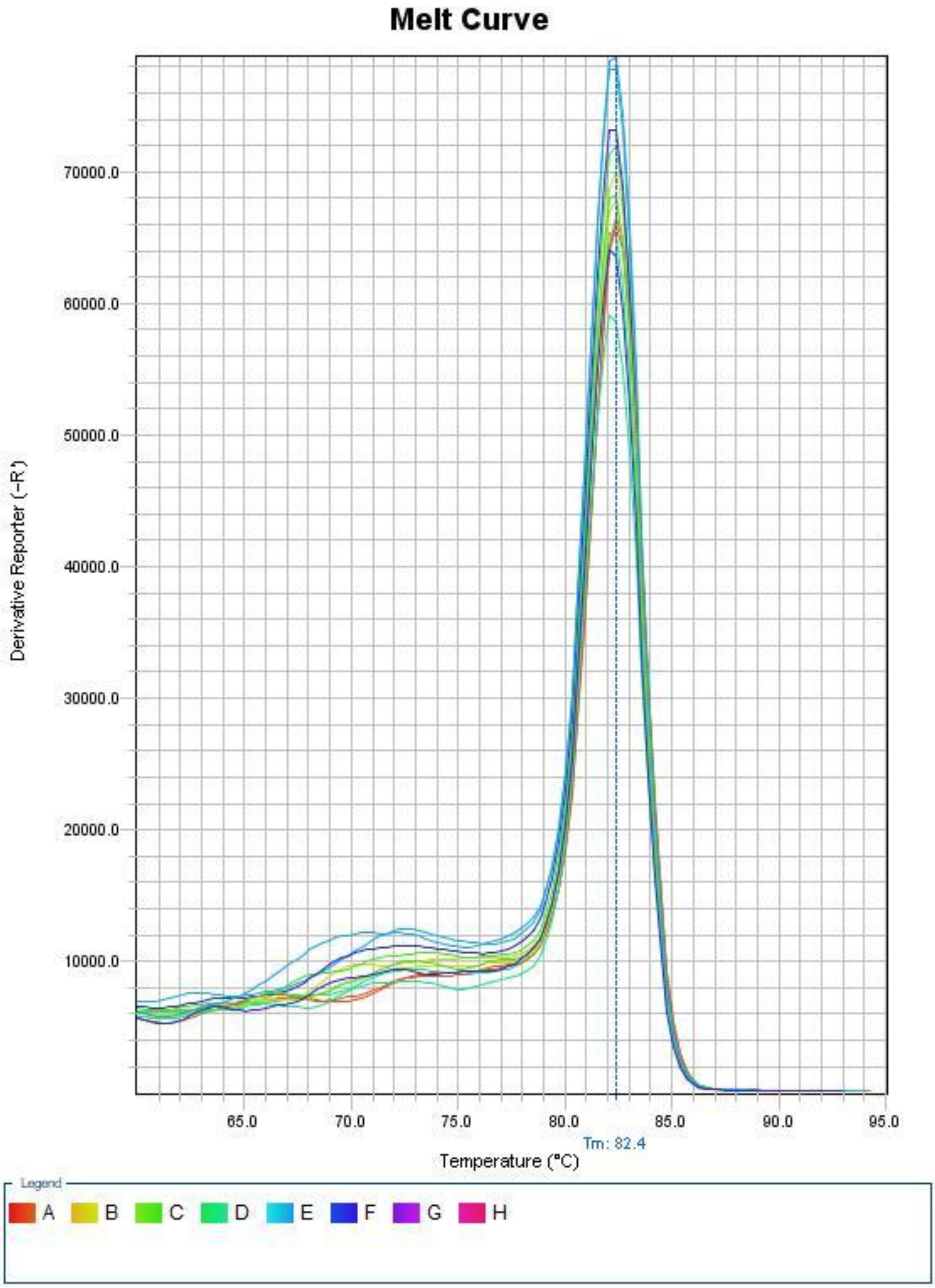

Melt Curve zo-1
